# Integrative analysis of mRNA stability regulation uncovers a metastasis-suppressive program in breast cancer

**DOI:** 10.1126/sciadv.aea9061

**Published:** 2026-03-11

**Authors:** Heather Karner, Tabea C. Mittmann, Vicky W. Chen, Ashir A. Borah, Andreas Langen, Hassan Yousefi, Lisa Fish, Balyn W. Zaro, Albertas Navickas, Hani Goodarzi

**Affiliations:** ^1^ Department of Biochemistry and Biophysics, University of California San Francisco, San Francisco, CA, USA.; ^2^ Department of Urology, University of California San Francisco, San Francisco, San Francisco, CA, USA.; ^3^ Helen Diller Family Comprehensive Cancer Center, University of California, San Francisco, San Francisco, CA, USA.; ^4^ Faculty of Medicine, University of Münster, Münster, Germany.; ^5^ Department of Genetics, Stanford University, Palo Alto, CA, USA.; ^6^ Biological and Medical Informatics Graduate Program, University of California, San Francisco, San Francisco, CA, USA.; ^7^ Arc Institute, Palo Alto, CA, USA.; ^8^ Department of Pharmaceutical Chemistry, University of California, San Francisco, San Francisco CA, USA.; ^9^ Cardiovascular Research Institute, University of California, San Francisco, San Francisco, CA, USA.

## Abstract

Heterogeneity in cancer gene expression is typically linked to genetic and epigenetic alterations, yet the extent of contribution from posttranscriptional regulation remains unclear. Here, we systematically measured messenger RNA (mRNA) dynamics across diverse breast cancer models, revealing that mRNA stability substantially shapes gene expression variability. To decipher these dynamics, we developed GreyHound, an interpretable multimodal deep-learning framework integrating RNA sequence features and RNA binding protein (RBP) expression. GreyHound identified an extensive network of RBPs and their regulons underlying variations in mRNA stability, including a regulatory axis centered on RBP RBMS3 and redox regulator TXNIP. *RBMS3* depletion resulted in targeted transcript destabilization—associated with poor clinical outcomes and enhanced metastatic potential in xenograft models. In vivo epistasis studies confirmed that RBMS3-mediated regulation of *TXNIP* mRNA stability drives this metastasis-suppressive program. These findings identify a key posttranscriptional mechanism in breast cancer and illustrate how interpretable models of RNA dynamics can uncover regulatory programs in disease.

## Teaser

A deep-learning model links RNA stability control to breast cancer metastasis via a key posttranscriptional regulatory mechanism.

## INTRODUCTION

Precise regulation of gene expression is essential for maintaining cellular identity and function, and its disruption is a hallmark of cancer. While genetic and epigenetic changes are well-established drivers of transcriptomic variability in tumors, accumulating evidence indicates substantial contributions from posttranscriptional mechanisms (*1*). In breast cancer, where metastasis is the primary cause of mortality (*2*), these changes are often driven by alterations in gene regulatory networks. Despite growing recognition of posttranscriptional regulation’s impact on cancer, the extent to which mechanisms such as mRNA stability contribute to gene expression variation across cancer transcriptomes remains unexplored (*3*, *4*). Recent work by our group and others has underscored the importance of posttranscriptional mechanisms in cancer progression (*5*–*7*), highlighting the need for a more systematic and quantitative assessment of these regulatory processes in breast cancer.

RNA stability is regulated through the combined action of cis-acting RNA sequence elements and trans-acting factors such as RNA binding proteins (RBPs). On the cis side, specific sequence or structural motifs, often embedded within the transcript’s 3′ untranslated region (3′UTR), modulate transcript stability by providing direct binding sites (*8*–*10*). On the trans side, RBPs recognize and bind these motifs, interacting with other RBPs and effector proteins to form regulatory complexes (*11*). These regulatory modules then stabilize or destabilize targeted mRNA transcripts. Historically, cis and trans determinants have been studied separately, with interactions combined only post hoc (*8*, *12*–*14*). However, because multiple RBPs can recognize the same sequence element and vice versa, posttranscriptional regulation inherently involves many-to-many relationships. To better capture this complexity, we developed GreyHound, a computational framework that jointly integrates RNA sequence features and RBP expression data to predict transcript stability and identify candidate posttranscriptional regulatory networks. Unlike previous approaches, GreyHound enables simultaneous evaluation of both cis and trans determinants, providing a comprehensive view of RNA stability regulation. We trained GreyHound with RNA stability and expression profiling data from six breast cancer cell lines representing diverse breast cancer subtypes. Although transcript decay rates were broadly conserved across these cell lines, we observed notable variations contributing to cancer heterogeneity. Using GreyHound, we identified candidate cis-trans regulatory pairs that influenced transcript stability across these breast cancer models. The regulatory interaction between the RBP RBMS3 (RNA binding motif single-stranded interacting protein 3) and its associated sequence element emerged as the most significant regulatory axis.

Through loss- and gain-of-function studies, we confirmed RBMS3 as a key posttranscriptional regulator of mRNA stability. Consistently, reduced expression of *RBMS3* was strongly associated with disease progression and aggressive clinical phenotypes in patient cohorts. Analysis of the RBMS3 target regulon revealed significant enrichment for metastasis-related biological pathways, prompting us to investigate metastatic potential downstream of this regulatory program. Overexpression of *RBMS3* reduced metastatic potential and its knockdown enhanced metastatic lung colonization across independent xenograft models of breast cancer. Using a systematic in vivo CRISPR-interference screen, we identified TXNIP (thioredoxin-interacting protein) as a critical downstream target of RBMS3; depletion of *TXNIP* resulted in similar phenotypes as *RBMS3* loss-of-function phenotypes, and subsequent in vivo lung colonization experiments confirmed the epistatic relationship between RBMS3 and TXNIP. Collectively, our study uncovered a previously unknown metastasis-suppressive regulatory program mediated through posttranscriptional stabilization of a specific regulon, demonstrating the capability of our computational model, GreyHound, to explore clinically relevant post-transcriptional regulatory networks in cancer.

## RESULTS

### Heterogeneity in mRNA dynamics across cell line models of breast cancer

**Fig. 1.**
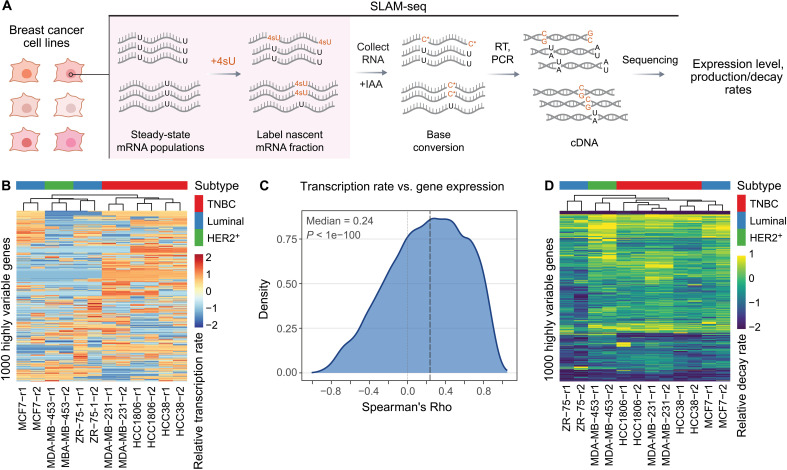
SLAM-seq reveals heterogeneity in mRNA transcription and stability in models of breast cancer. (**A**) Schematic of SLAM-seq showing how 4-thiouridine (4sU) labeling causes T to C nucleotide conversions during library preparation and are detected during analysis of sequencing data to determine metabolic labeling rate. This in conjunction with that expression data can be used to estimate mRNA decay in cell lines. (**B**) Normalized transcription rates based on observed C/T ratios from SLAM-seq; shown are the top thousand highly variable genes in this dataset. Note that the columns are clustered using hierarchical clustering and biological replicates group together. (**C**) A density plot showing the distribution of the Spearman correlations between SLAM-based transcription rates and expression for each gene across samples. Wilcoxon signed-rank test was used. (**D**) A heatmap of normalized mRNA decay rate estimates across cell lines and replicates for 1000 highly variable genes. [(B) and (D)] Normalization of transcription and decay rates using *z*-scores per row.

To systematically characterize RNA dynamics across different breast cancer models and subtypes, we performed metabolic RNA labeling using SLAM-seq [thiol(SH)–linked alkylation for the metabolic sequencing of RNA] (*15*) on six breast cancer cell lines representing three major subtypes: luminal (ZR-75-1, MCF7), HER2-positive (MDA-MB-453), and triple-negative breast cancer (TNBC; HCC1806, MDA-MB-231, and HCC38) (Fig. 1A). To visualize transcription-rate heterogeneity across the cell lines, we examined transcription rates for the top 1000 highly variable genes (Fig. 1B). As expected, transcription rates largely correlated with gene expression across all genes, explaining a substantial fraction of gene expression variation (Fig. 1C). However, a notable portion of gene expression variability remained unexplained by transcriptional changes alone (Fig. 1C), suggesting that additional regulatory mechanisms contribute to variations in mRNA abundance. We hypothesized that posttranscriptional control, particularly differential RNA decay rates, likely account for the remainder of this variation.

We used gene expression values and metabolic labeling rates from SLAM-seq data to estimate mRNA decay rates in each sample. These decay rate measurements were highly reproducible across biological replicates (average Pearson *R* ≈ 0.9; fig. S1A), validating the robustness of our profiling approach. While global mRNA decay patterns were broadly conserved across cell lines (average pairwise *R* = 0.59), we identified substantial heterogeneity in decay rates for hundreds of transcripts (Fig. 1D). Principal components analysis of these decay profiles revealed subtype-specific structures as well, with TNBC models clustering together (fig. S1B) while biological replicates show the highest pairwise correlation among samples (fig. S1C). Consistently, analysis of variance (ANOVA) revealed ~100 genes with statistically significant cell line-specific decay patterns [*P* < 0.003, false discovery rate (FDR) < 0.25], reflecting meaningful posttranscriptional differences across breast cancer subtypes (fig. S1D). These findings motivated us to explore the underlying determinants of variability in RNA stability, which we describe in subsequent sections.

### Integrative modeling of mRNA stability reveals posttranscriptional heterogeneity in breast cancer

**Fig. 2.**
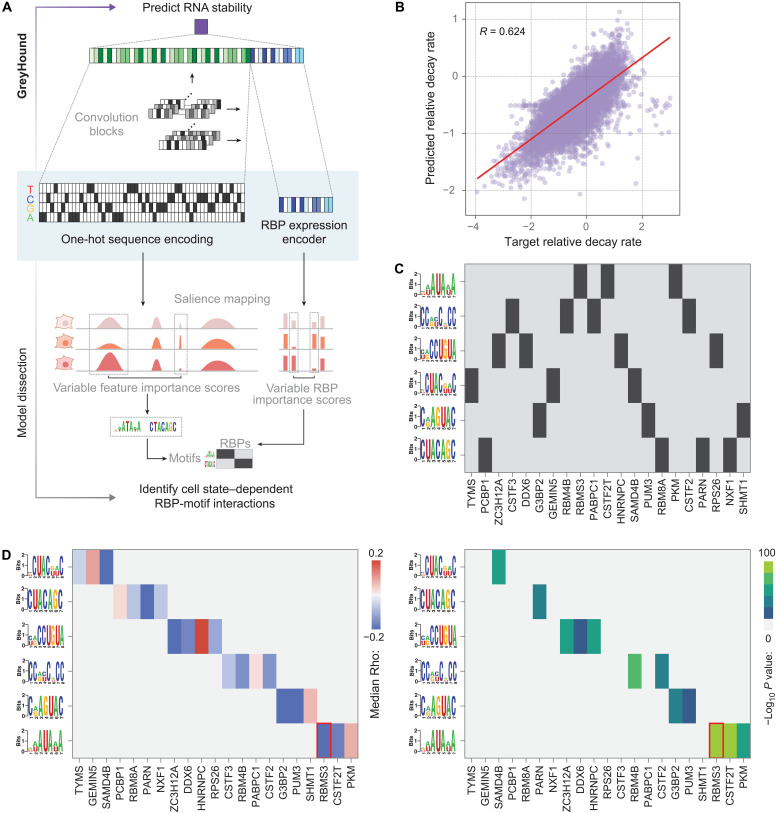
GreyHound reveals the cis and trans posttranscriptional regulatory programs in breast cancer cells. (**A**) Our strategy for measuring feature importance using model gradients at input. As denoted here, we specifically focused on features that show differential importance across cell lines. (**B**) Performance of our GreyHound model on a held-out test set. Each point represents the measurement and prediction for a given gene and a given cell line; Pearson correlation is shown. (**C**) A map of putative RBP-motif interaction revealed by motif enrichment analysis among the sequences attributed to each RBP of interest. (**D**) Further evaluation of these interactions by analyzing the distribution of Spearman correlations between the expression of each RBP and the decay rate measurements for its putative target transcripts based on motif occurrence. Shown are the median Spearman correlation for each nominated RBP-regulon pair (left) and its associated *P* value calculated using Wilcoxon rank sum test (right). The interaction between RBMS3 and an AUA element is highlighted (red box) as the most significant interaction.

While mRNA decay rates are similar across breast cancer cell lines, there are also notable cell-state specific variations. To understand the role of posttranscriptional control that gives rise to (and may be influenced by) heterogeneity in breast cancer, we sought to reveal the cis-trans regulatory interactions that underlie the differential regulation of mRNA stability across breast cancer lines. To comprehensively capture the complexity of RNA stability regulation across diverse cellular contexts, we developed GreyHound, a deep-learning framework that jointly integrates RNA sequence features and RBP expression data (Fig. 2A). Unlike previous approaches, GreyHound simultaneously evaluates both cis and trans determinants of transcript stability, enabling the identification of context-dependent posttranscriptional regulatory interactions. The model architecture uses convolutional layers for sequence processing coupled with a pretrained variational autoencoder (VAE) for RBP expression encoding (fig. S2, A and B). As shown in Fig. 2B, given the expression profile of RBPs in a cell and the mRNA sequence of interest, GreyHound predicts the estimated decay rate for that mRNA within the cellular context defined by its RBP expression profile. Evaluating the performance of this model on a held-out test set of RBP-gene pairs across cell lines showed a general agreement between the predicted and measured mRNA decay rates (*R* = 0.62).

To decipher the cell state–dependent regulatory logic learned by the model, we implemented a comprehensive model dissection approach that combines gradient-based saliency analysis with rigorous statistical validation (Fig. 2A). For each gene-cell line pair, we estimated feature importance across the sequence as well as RBPs by calculating gradient at input (i.e., akin to saliency). For both heads of the model, we specifically focused on features whose importance shows variation across cell states. For sequence analysis, we identified regions of variable importance across cell lines by modeling the mean-variance relationship and identifying deviations from the expected variance. We used a smoothened coefficient of variation (CV) across each sequence to mark highly variable regions (top 10% of CV). These regions were merged if they occurred within 20 nucleotides of each other, defining key regulatory regions relevant to cell state–dependent predictions of the model. In parallel, for RBP inputs, we similarly identified proteins with consistently highly variable importance scores across conditions. To pinpoint the underlying cis-regulatory elements overrepresented across the found highly variable regions, we used FIRE (finding informative regulatory elements) to perform de novo motif discovery (*13*). To link these sequence motifs to the selected RBPs, we performed a pair-wise enrichment analysis for each motif among the sequences for which the RBP was identified as being differentially salient (Fig. 2C and fig. S2C). The statistical significance of these RBP-motif interactions was further validated through Spearman correlation analysis between RBP expression and target transcript stability (Fig. 2D). The most significant interaction within this predicted network of posttranscriptional regulatory interactions was between RBMS3 and an AUA-rich sequence element, highlighted by red boxes in Fig. 2D. As shown in fig. S2D, the decay rates measured across cell lines for the RBMS3 regulon, defined as the set of transcripts that carry the identified AUA elements, were on average anticorrelated with the expression of this RBP in these lines. Our RBP-motif interaction map described here provides a quantitative framework for understanding posttranscriptional regulation of RNA stability across cell states and nominates RBMS3 as a potential regulator of mRNA stability in the context of breast cancer.

### RBMS3 acts as a posttranscriptional regulator of mRNA stability in breast cancer cells

**Fig. 3.**
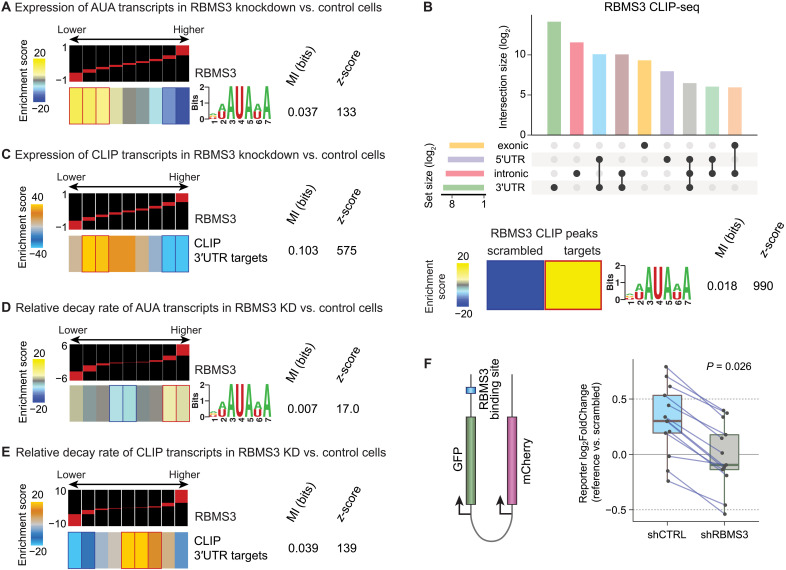
RBMS3 binding affects target regulon expression and stability in breast cancer cells. (**A**) Enrichment and depletion patterns of AUA element containing the transcript’s expression level in breast cancer cell line MDA-MB-231 with *RBMS3* knocked down versus control cells. (**B**) RBMS3-binding site locations identified by CLIP-seq (top). Enrichment of the AUA motif among RBMS3 CLIP-seq–binding sites (bottom). (**C**) Enrichment and depletion patterns of RBMS3 target regulon expression in breast cancer cell line MDA-MB-231 with *RBMS3* knocked down versus control cells. (**D**) Enrichment and depletion patterns of RBMS3 target AUA motif–containing transcripts relative RNA decay rate in breast cancer cell line MDA-MB-231 with *RBMS3* knocked down versus control cells. (**E**) Enrichment and depletion patterns of RBMS3 target regulon relative RNA decay rate in breast cancer cell line MDA-MB-231 with *RBMS3* knocked down versus control cells. (A and C to E) Shown are enrichment patterns along with calculated MI and associated *z*-score. See (*13*) for details. (**F**) Dual reporter system that expresses mCherry and GFP driven by a bidirectional CMV lentiviral promoter. RBMS3-binding recognition sites are inserted into the 3′UTR of GFP (left). Expression analysis of paired target sequence to SCR control sequence in MDA-MB-231 *RBMS3* knockdown versus control cells. Statistical significance was assessed using a paired Wilcoxon signed-rank test (right).

Following the identification of RBMS3 as a potential regulator of mRNA stability in breast cancer by our GreyHound model, we next sought to investigate the functional impact of *RBMS3* expression on its target regulon. To this end, *RBMS3* was silenced in the MDA-MB-231 breast cancer cell line, which exhibits relatively high endogenous *RBMS3* expression compared to other breast cancer cell lines, using two independent short hairpin RNAs (shRNAs). Successful knockdown of *RBMS3* was confirmed before an RNA sequencing (RNA-seq) analysis to assess gene expression changes resulting from *RBMS3* depletion (fig. S3A). Motif enrichment analysis using FIRE (*13*) revealed a strong and highly significant overrepresentation of the AUA motif within the 3′UTR of transcripts that were down-regulated in *RBMS3* knockdown cells (Fig. 3A). To ensure that this observation was not an artifact of motif selection, we used RBMS3-binding scores from DeepBind (D00138.001) to identify transcripts that are likely targets of RBMS3 based on its binding preferences learned from RNA compete assays (*16*). As shown in fig. S3B, the DeepBind-predicted RBMS3 regulon were also significantly down-regulated upon *RBMS3* knockdown, supporting the hypothesis that RBMS3 affects the stability of its target transcripts.

To further validate our findings regarding RBMS3 and its putative regulon, we experimentally identified the RBMS3-bound target transcripts using a modified version of CLIP-seq (cross-linking and immunoprecipitation sequencing) (*17*, *18*). Since reliable antibodies for immunoprecipitation of endogenous RBMS3 under CLIP conditions were not available, we expressed FLAG-tagged RBMS3 in MDA-MB-231 cells to perform transcriptome-wide mapping of its binding sites. Consistent with our computational predictions, we observed (i) extensive RBMS3 binding within 3′UTRs across thousands of sites and (ii) a significant enrichment of the AUA motif previously identified through GreyHound analysis (Fig. 3B). De novo motif discovery among the RBMS3 CLIP–binding sites also identified an AUA element, providing evidence for in vivo binding of RBMS3 to this motif (fig. S3C). Furthermore, when we repeated our analysis from the previous section with this experimentally defined set of RBMS3-bound targets instead of the motif-based putative regulon, we found that these transcripts were also significantly down-regulated in *RBMS3* knockdown cells (Fig. 3C). These findings provide strong evidence that RBMS3 binds and stabilizes its target transcripts through interactions with AUA-containing elements in their 3′UTRs.

To ensure that the observed reduction in transcript abundance in response to *RBMS3* silencing is due to posttranscriptional changes in mRNA stability and not transcriptional modulations, we performed SLAM-seq (*15*) in MDA-MB-231 cells following *RBMS3* knockdown. Consistent with our previous observations, transcripts exhibiting higher decay rates upon *RBMS3* depletion were significantly enriched for the 3′UTR-AUA motif (Fig. 3D). However, the AUA motif did not significantly affect differential transcriptional measurements: Transcripts containing this element were neither enriched nor depleted in transcriptional output (fig. S3D), indicating that RBMS3’s primary mode of action is posttranscriptional rather than transcriptional. To confirm the robustness of these findings across different target definitions, we replicated the analysis using both the DeepBind-predicted regulon (fig. S3E) and the RBMS3 CLIP–derived target set (Fig. 3E). In each case, transcripts bearing the AUA motif exhibited significantly increased decay rates upon *RBMS3* depletion.

To directly test whether RBMS3-binding sites are sufficient and required for RBMS3-mediated regulation of mRNA stability, we used a dual-reporter system previously established for investigating posttranscriptional regulatory elements in living cells (*9*, *19*). From a set of high-confidence RBMS3-bound 3′UTRs (described in later sections), we selected representative binding sites for functional validation. These sequences were cloned downstream of a green fluorescent protein (GFP) coding region in our dual-reporter vector, along with matched scrambled (SCR) control sequences that preserved dinucleotide composition but disrupted motif content.

This reporter library was transduced into MDA-MB-231 control and *RBMS3* knockdown cells, and targeted RNA-seq was used to quantify reporter expression as a readout of cis-element activity and its dependence on RBMS3. We made two key observations consistent with our molecular model of RBMS3 action: (i) In most cases, the presence of the RBMS3-binding site increased reporter mRNA levels relative to SCR controls (fig. S3F), and (ii) this effect was absent in *RBMS3*-depleted cells, indicating that the observed stabilization was dependent on *RBMS3* expression (Fig. 3F). Together, these findings demonstrate that direct RBMS3-RNA interactions are both necessary and sufficient to confer increased transcript stability.

To assess whether GreyHound’s regulatory predictions extend beyond RBMS3, we analyzed the effect of knockdown for two additional high-ranked RBPs—*SAMD4B* and *RBM4B*. For both proteins, the GreyHound-inferred motifs were significantly enriched among transcripts whose stability changed upon knockdown, although the effect sizes were weaker than those observed for RBMS3 (fig. S3G). These results indicate that GreyHound captures regulatory signals for multiple RBPs while highlighting RBMS3 as the most prominent prediction.

### RBMS3 and its downstream target regulon are associated with breast cancer progression

**Fig. 4.**
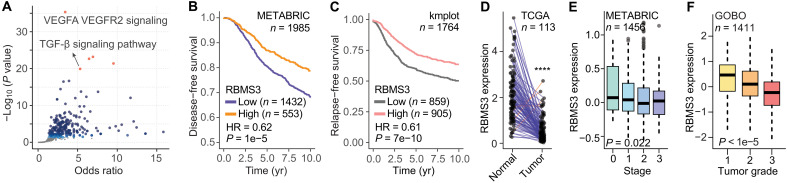
RBMS3 is a predictor of breast cancer progression. (**A**) Gene set enrichment analysis using Appyters (*20*) to identify pathways that are enriched among the transcripts bound by RBMS3 based on CLIP-seq annotations. Vascular endothelial growth factor (VEGF) and transforming growth factor–β (TGF-β) pathways were the top associated gene sets. (**B**) Analysis of disease-free survival in the METABRIC cohort (*21*) relative to *RBMS3* expression. (**C**) Meta-analysis of relapse-free survival in smaller published cohorts (*22*) relative to *RBMS3* expression. (B and C) The Mantel-Cox test was used to assess significance. Low *RBMS3* expression is indicative of poor prognosis for disease-free and relapse-free survival in patients with breast cancer. (**D**) Analysis of paired normal and breast cancer samples in the TCGA dataset revealed an almost universal reduction in *RBMS3* expression in tumor tissue. Paired Mann-Whitney test was used, ****P < 0.0001. (**E**) *RBMS3* expression in METABRIC cohort (*21*) by tumor stage (0 to 1, early; 2 to 3, late). Mann-Whitney *U* test was used for statistical comparison. (**F**) *RBMS3* expression across 1411 tumor samples stratified on the basis of their annotated grades (GOBO meta-analysis) (*38*). ANOVA was performed. [(E) and (F)] Bars show means, interquartile ranges, and SEM. yr., year.

As we have demonstrated in the preceding sections, RBMS3 modulates the stability of its target mRNA transcripts. Next, we wanted to delineate which downstream molecular and cellular pathways are affected by RBMS3. Therefore, we conducted a series of gene set enrichment analyses. First, we used Appyters (*20*) to identify pathways that are enriched among the transcripts bound by RBMS3 based on CLIP-seq annotations. As shown in Fig. 4A, we identified vascular endothelial growth factor and transforming growth factor–β pathways as the top associated gene sets. Parallel analyses of gene and protein expression changes in response to *RBMS3* knockdown similarly identified gene sets associated with breast cancer progression and metastasis (fig. S4, A and B). These results raised the possibility that RBMS3 plays a role in breast cancer progression. Specifically, our findings indicate that *RBMS3* silencing is associated with increased expression of oncogenic and prometastatic pathways.

We next sought to evaluate the association between *RBMS3* expression and clinical outcomes in breast cancer using patient data from several independent breast cancer datasets. Analysis of the METABRIC dataset (*21*), containing gene expression profiles for ~2000 patients, revealed that diminished *RBMS3* levels were indicative of poor clinical outcomes (Fig. 4B). Data from a meta-analysis of existing breast cancer datasets (*22*) likewise revealed a negative association between *RBMS3* expression and relapse-free survival (Fig. 4C). A negative association of *RBMS3* expression and progression-free survival (PFS) was also observed in the analysis of both RBMS3 mRNA and protein levels in the TCGA-BRCA and TCGA-BRCA-CPTAC breast cancer cohorts (fig. S4C). In addition, *RBMS3* expression was consistently reduced in higher-grade and later-stage tumors across several independent breast cancer cohorts (Fig. 4, D to F). These findings are consistent with prior research that analyzed *RBMS3* expression in patient data from the TCGA dataset and similarly reported that reduced *RBMS3* expression is associated with poor clinical outcomes (*23*). Together, these results demonstrate a significant association between *RBMS3* loss and adverse clinical outcomes, highlighting its potential utility as a prognostic biomarker in breast cancer.

Last, we observed that the negative association observed between *RBMS3* expression and survival was largely attributed to the basal and claudin-low subtypes in the METABRIC cohort (fig. S4, D and E) (*21*). On the basis of this observation—and given the known increased metastatic potential of these subtypes—we focused subsequent functional and phenotypic analyses specifically on the triple-negative models of breast cancer.

### *RBMS3* modulation affects breast cancer metastasis and invasion

**Fig. 5.**
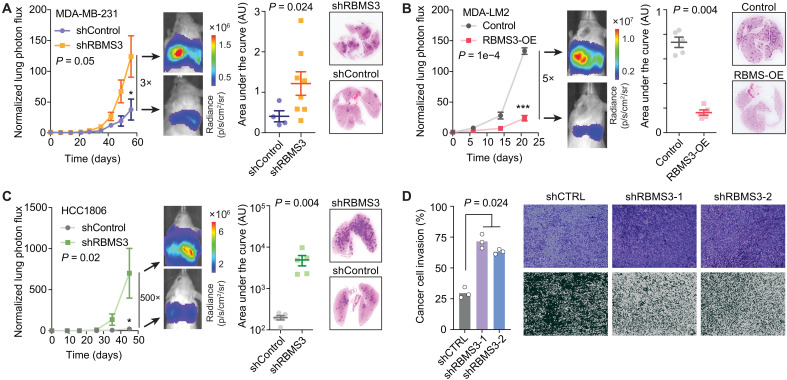
Loss of *RBMS3* increases metastatic lung colonization and invasion phenotypes. (**A** to **C**) Lung colonization assay for control and either *RBMS3* knockdown or *RBMS3* overexpression. Luciferase-labeled cells were injected via tail vein, and their metastatic growth in the lungs was measured over time. The magnitude of the signal, shown here as a heatmap for a representative mouse from each cohort, reflects the metastatic burden. Bars on time-course plots show means ± SEM, and ANOVA was performed (left). Also included are area under the curve (AUC) of log-normalized signal in the lungs of mice over 25 days. Summary overlay shows means ± SEM and Mann-Whitney *U* test used to determine statistical significance (middle). Also, hematoxylin and eosin–stained lung sections for exemplary mice (right). (A) Lung colonization assays of control (*N* = 4 biological replicates) and *RBMS3* knockdown MDA-MB-231 cells (*N* = 4 biological replicates). (B) Lung colonization assays for control (*N* = 5 biological replicates) and *RBMS3* overexpression MDA-LM2 cells (*N* = 5 biological replicates). (C) Lung colonization assays for control (*N* = 5 biological replicates) and *RBMS3* knockdown HCC1806 cells (*N* = 5 biological replicates). (**D**) Invasion assays for control and *RBMS3* knockdown MDA-MB-231 cells. Images are representative (i.e., median) in each group. In processed images, black is empty space and white is cells. The graph shows fraction that is white (i.e., % area covered), and the Mann-Whitney *U* test was used for statistical comparison. *P < 0.05; ***P < 0.001.

To establish a causal link between *RBMS3* silencing and higher metastatic potential, we performed lung colonization assays in immunodeficient NSG [nonobese diabetic (NOD) scid gamma] mice. We injected MDA-MB-231 cells with *RBMS3* knocked down (two independent shRNAs) or control cells via tail vein and measured metastatic burden in the lungs over time using in vivo imaging (Fig. 5A). Consistent with our earlier findings, mice injected with *RBMS3* knockdown cells exhibited significantly greater metastatic lung colonization compared to those injected with control cells. We also established that this increase is not attributable to higher proliferation rate in these cells (fig. S5A).

To complement this loss-of-function approach, we performed a gain-of-function experiment, by overexpressing *RBMS3* in the MDA-LM2 cell line, a highly metastatic derivative of MDA-MB-231 (*24*). Elevated *RBMS3* expression resulted in a marked reduction in lung colonization (Fig. 5B) again independent of the proliferation rates of these cells (fig. S5B). To ensure that our findings are not limited to the MDA-MB-231 background, we also included the HCC1806 cell line as an independent model of claudin-low breast cancer (*25*). Consistent with our prior finding, shRNA-mediated silencing of *RBMS3* led to a substantial and significant increase in metastatic lung colonization in xenografted NSG mice with no changes in proliferation rates (Fig. 5C and fig. S5C).

Given the absence of proliferation differences, and considering that metastasis is also influenced by cancer cell invasiveness, we next assessed whether RBMS3 affects invasive behavior using transwell invasion assays. *RBMS3* knockdown in MDA-MB-231 cells led to a significant increase in invasive capacity (Fig. 5D), indicating that RBMS3 plays an important role in regulating this key hallmark of breast cancer metastasis (*2*). Collectively, these results establish RBMS3 as a suppressor of metastasis and invasion in claudin-low/basal breast cancers.

### The RBMS3-TXNIP regulatory axis suppresses breast cancer metastasis

Our results so far indicate that (i) RBMS3 functions as a regulator of mRNA stability, (ii) it is associated with breast cancer progression in basal-like/claudin-low subtypes, and (iii) *RBMS3* expression suppresses metastatic lung colonization in cell line–derived xenograft models. However, to confirm that RBMS3’s metastasis-suppressive role is connected to its function as a posttranscriptional regulator and to better understand the molecular mechanisms downstream of RBMS3, we sought to identify its target transcripts that contribute to metastasis. To select these candidate genes, we focused on genes that show direct RBMS3 binding in their 3′UTRs. We observed that for these genes, the changes in gene expression in response to *RBMS3* silencing is overall correlated at the mRNA and protein levels (Spearman correlation of 0.2, *P* = 2 × 10^−19^). Among these genes, we selected those that were significantly down-regulated in both datasets (logFC < −0.25 and *P* value < 0.05). We also selected transcripts with no reduction in transcription rate based on our SLAM-seq data. Last, we also performed survival analyses in the METABRIC dataset (*21*) and selected those genes that are associated with better survival outcomes. These criteria resulted in the selection of 13 genes.

**Fig. 6.**
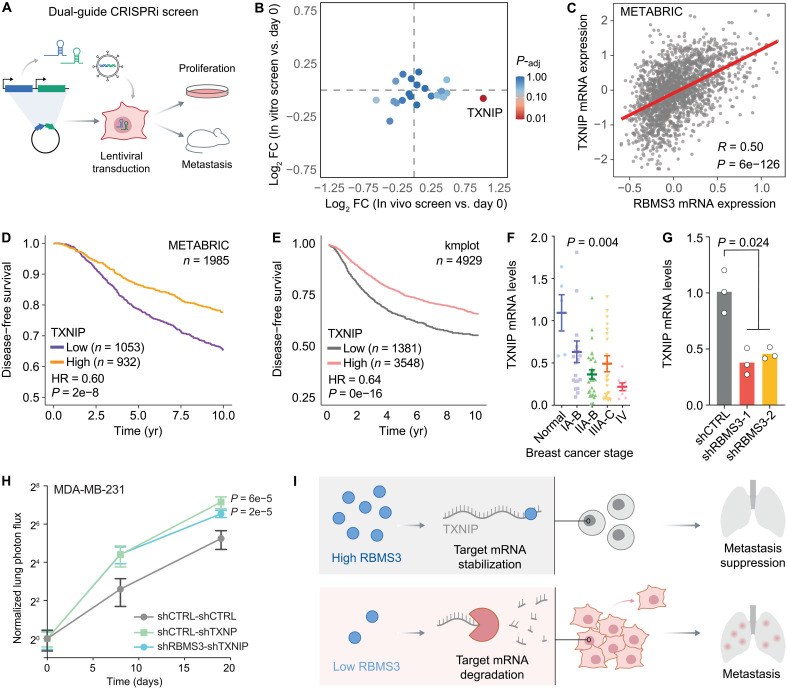
*TXNIP* knockdown shares phenotype with RBMS3 and acts as a tumor suppressor. (**A**) Schematic of the dual-guide CRISPR interference (CRISPRi) screen. (**B**) Analysis of CRISPRi screen comparing the abundance of cells expressing each guide between in vivo– and in vitro–grown cells with DESeq2. *TXNIP* was observed to be present at high levels in vivo and low levels in vitro indicating high metastasis but low proliferation. (**C**) Comparative analysis of *TXNIP* to *RBMS3* expression in the METABRIC cohort (*21*). Shown is Pearson correlation with *t* statistic for nonzero correlation. (**D**) Analysis of disease-free survival in the METABRIC cohort (*21*) relative to *TXNIP* expression. (**E**) Meta-analysis of relapse-free survival in smaller published cohorts (*22*) relative to *TXNIP* expression. (D and E) The Mantel-Cox test was used to measure significance. Low *TXNIP* expression is indicative of poor prognosis for disease-free survival in patients with breast cancer. (**F**) The expression levels of *TXNIP* in 90 tumor samples at different stages of breast cancer was measured by qPCR; bars show means and SEM. ANOVA was performed. (**G**) The expression levels of *TXNIP* in *RBMS3* knockdown MDA-MB-231 cells and control cells was measured by qPCR and compared using Mann-Whitney *U* test. (**H**) Lung colonization assays of control (*N* = 5 mice), *TXNIP* knockdown (*N* = 5 mice), and *RBMS3-TXNIP* double knockdown (*N* = 5 mice) in MDA-MB-231 cells. Luciferase-labeled cells were injected via tail vein, and their metastatic growth in the lungs was measured over time. ANOVA was performed; bars indicate means and SEM. (**I**) Molecular mechanism of RBMS3 metastasis suppression through posttranscriptional regulatory action.

Because of this set being too large for individual functional validation in xenograft models, we instead opted to conduct an in vivo–pooled CRISPR-interference screen in NSG mice. We used the dual-guide system, where two guides are simultaneously cloned for each target gene to ensure effective knockdown (*26*). MDA-MB-231 cells were transduced with the guide library that included 10 nontargeting controls, and these cells were then injected into mice tail vein (*N* = 3). In parallel, we also collected in vitro–proliferated samples to account for potential changes in proliferation rates and to focus on the in vivo metastasis phenotype observed for RBMS3. Once lungs were colonized and metastatic foci were formed, we extracted the lungs of the mice and isolated genomic DNA (Fig. 6A). We then used targeted polymerase chain reaction (PCR) for dual-guide libraries and high-throughput sequencing to quantify the abundance of cells expressing each guide within the in vivo– and in vitro–grown cells. Among the 13 candidates, TXNIP emerged as showing a significant lung colonization phenotype without impact on proliferation (Fig. 6B). This phenotype mirrors that of *RBMS3* knockdown, suggesting that TXNIP may be a downstream effector of RBMS3 in suppressing metastasis.

Consistent with this model, we observed a significant and positive correlation between *RBMS3* and *TXNIP* expression across multiple datasets, namely METABRIC (*21*), TCGA, and MBCProject (Fig. 6C and fig. S6, A and B). Similarly, we also observed that lower expression of *TXNIP* is also strongly associated with poor outcomes (Fig. 6, D and E). To independently validate this, we measured *TXNIP* expression in a cohort of 90 breast tumor samples across different disease stages and found a significant association between *TXNIP* silencing and breast cancer progression (Fig. 6F).

To confirm that TXNIP acts as a downstream target of RBMS3, we measured its pre-mRNA levels in *RBMS3* knockdown cells using quantitative PCR (qPCR). Consistent with our previous observation of a ~twofold reduction in mRNA (*P* = 1 × 10^−3^) and protein levels (*P* = 0.047) of TXNIP in *RBMS3* knockdown cells, silencing *RBMS3* resulted in a significant reduction in *TXNIP* mRNA levels (fig. S6, C and G). However, we observed no change in *TXNIP* pre-mRNA levels upon *RBMS3* knockdown (fig. S6D), indicating that RBMS3 does not influence *TXNIP* transcription rates.

To further investigate the functional relationship between RBMS3 and TXNIP in vivo, we performed an epistasis experiment using MDA-MB-231 cells engineered to express a dual shRNA cassette targeting both *RBMS3* and *TXNIP*. As shown in Fig. 6H, knockdown of *TXNIP* alone significantly increased metastatic lung colonization relative to control cells, consistent with its role as a metastasis suppressor. Cosilencing of *RBMS3* and *TXNIP* also enhanced metastatic colonization relative to controls. However, the extent of colonization in the double knockdown condition was not greater than that observed with *TXNIP* knockdown alone (Fig. 6H). If RBMS3 and TXNIP acted through independent pathways, we would expect the double knockdown to yield an additive or synergistic increase in metastasis. Instead, the lack of an additive effect supports a model in which RBMS3 acts upstream to promote *TXNIP* expression.

## DISCUSSION

Our study identifies the RBMS3-TXNIP posttranscriptional regulatory axis as a key suppressor of breast cancer metastasis. We demonstrate that RBMS3 binds and stabilizes the *TXNIP* transcript and that loss of *RBMS3* reduces *TXNIP* levels, leading to enhanced metastatic colonization without affecting proliferation (Fig. 6I). This highlights a molecular mechanism in which RBPs can exert metastasis-suppressive effects through regulation of transcript stability.

TXNIP has been previously implicated as a tumor suppressor in several cancer types (*27*), including breast cancer (*28*, *29*), although its regulation at the posttranscriptional level remains poorly understood. We show that RBMS3 is a direct, positive regulator of *TXNIP* mRNA stability, providing previously unknown mechanistic insight into how TXNIP is controlled. Notably, epistasis experiments confirmed that RBMS3 requires TXNIP to mediate its antimetastatic effects, reinforcing the functional relevance of this regulatory axis.

The discovery of this pathway was enabled by our development of GreyHound, a computational framework that integrates RNA sequence and RBP expression data to infer regulators of transcript stability. GreyHound analysis pinpointed RBMS3 as a candidate stabilizer of AUA-containing transcripts. Experimental validation of this prediction confirmed RBMS3’s role in stabilizing *TXNIP* and suppressing metastasis.

This work expands the biological roles of RBMS3 beyond its prior characterization as a c-MYC–related RBP (*30*). Our data indicate that it acts as a broad regulator of mRNA stability with a particular role in constraining metastatic progression. These findings align with recent studies proposing tumor-suppressive roles for RBMS3 (*23*) and position it within the growing landscape of posttranscriptional regulators that influence metastasis. Several studies have linked RBMS3 to cancer cell–state regulation and phenotypes associated with invasion and progression. Block and colleagues identified RBMS3 as a common effector of epithelial-mesenchymal transition (EMT)-associated programs and reported that RBMS3 can modulate mRNA stability, including stabilization of the EMT transcription factor PRRX1 (*31*). The reported phenotypic consequences of RBMS3 perturbation have varied across studies and experimental contexts, consistent with model-dependent effects on invasive and metastatic behaviors (*31*, *32*).

Our study extends these observations by defining a mechanistically grounded metastasis-suppressive pathway centered on RBMS3-dependent stabilization of *TXNIP*. By integrating transcript stability modeling with direct RBMS3–RNA interaction mapping (CLIP-seq), functional epistasis demonstrating TXNIP requirement, and in vivo metastasis assays, we provide a rigorous framework linking RBMS3-mediated RNA regulation to metastatic colonization.

Because our decay measurements are derived from population-level SLAM-seq, the stability estimates represent averaged behaviors across heterogeneous cell states. As a result, potential variation in RNA stability arising from processes such as the cell cycle is not explicitly resolved. The intertumor heterogeneity captured by the six breast cancer cell lines reflects differences between established subtype models rather than single-cell–level variability. While these limitations define the resolution of our analysis, they do not alter the core conclusions regarding the RBMS3-TXNIP regulatory axis. Advances in single-cell metabolic labeling and stability modeling (*33*, *34*) may enable finer interrogation of state-dependent RNA decay programs.

More broadly, our approach provides a generalizable framework for uncovering regulatory pathways driven by mRNA stability. As transcriptome-wide mRNA stability measurements become more accessible, computational frameworks like GreyHound will enable systematic discovery of functional RNA-protein (RNP) interactions across diverse disease contexts.

In addition to defining the RBMS3-TXNIP axis as a metastasis-suppressive pathway, our findings suggest possible clinical relevance. Reduced *RBMS3* expression is associated with more aggressive disease, indicating potential prognostic value in identifying tumors with increased metastatic propensity. Although our data do not establish RBMS3 as a therapeutic target, they raise important questions about how RBMS3 becomes diminished in metastatic cells and whether restoring its regulatory activity could constrain metastatic progression. A deeper understanding of the mechanisms leading to *RBMS3* silencing, and whether they can be reversed, will be essential for determining whether this pathway can be leveraged in future clinical strategies.

## MATERIALS AND METHODS

### Genome-wide mRNA decay rate measurements across breast cancer cell lines

#### 4sU metabolic labeling of mRNA

One day before the SLAM-seq procedure (*15*), all cell lines were plated in biological replicates at 1 × 10^5^ cells per well on a 24-well plate. For 4-thiouridine (4sU) labeling, we incubated ZR-75-1, MDA-MB-453, HCC1806, MDA-MB-231, HCC38, and MCF7, as well as *RBMS3* knockdown and control MDA-MB-231 cells in their respective culture media supplemented with 4sU (100 μM) for 4 hours. After pulse labeling, the cells were washed twice with phosphate-buffered saline (PBS) and harvested directly in TRIzol Reagent (Invitrogen, 15596018). Total RNA was isolated and Iodoacetamide labeling was performed on all isolated RNA (input range 500 ng to 3 μg) using the SLAMseq Explorer and Kinetics Kits (Lexogen, 061), following the manufacturer’s instructions. Library preparation was carried out using the Quant-Seq 3’ mRNA-Seq Library Prep Kit (Lexogen, 016). Sequencing was performed on the HiSeq4000 with a SE65 run at the UCSF Center for Advanced Technologies.

#### Analysis of mRNA decay rate

We first used Salmon (v0.14.1) to align the reads to the human transcriptome (gencode v28). Transcripts with average expression >1 transcripts per million (TPM) across the cell lines were selected for further analysis (i.e., transcripts that are “expressed”). Bowtie2 (v2.3.5) was then used to align reads to these annotated major isoforms. Samtools mpileup command was used next to generate read stacks, which were then processed to count the number of T to C conversions. For every gene, we required the coverage to be at least 10 reads at counted Ts, filtered abundant variant calls (as they imply germline variant rather than chemical conversion), and finally calculated a C to T ratio (allowing for a pseudo-count of 1). We filtered outlier transcripts, using the 3 × interquartile range above or below median as thresholds. Note that biological replicates were combined at this stage by combining the counts before calculating the C to T ratio. Since the cells were pulsed with 4sU for 4 hours, 4sU incorporation, and therefore the resulting C to T ratio, are driven by the rate of transcription. At steady state, decay rate is equal to transcription rate normalized by the steady-state expression. So, we used this rule to estimate a decay rate (log transformed) for every gene across all cell lines, based on its SLAM-seq C to T ratio and its baseline expression (AU).

### Modeling context-specific mRNA stability using GreyHound

#### Data preparation and pre-processing

RNA expression data from breast cancer cell lines (HCC1806, MDA-MB-231, MCF7, MDA-MB-453, HCC38, and ZR-75-1) from above was normalized in TPM and log-transformed. Genes with low expression (sum of expression values across all samples ≤10) were filtered out, resulting in 12,388 genes for downstream analysis. RBPs were identified using the Gene Ontology term “RNA binding” (GO:0003723), which yielded 1378 RBPs that were present in our expression dataset. RNA decay estimates were similarly collected as described above.

For sequence data, we extracted the major isoform sequences from GENCODE v28, limiting our analysis to transcripts between 512 and 4096 nucleotides in length. This resulted in ~7000 transcripts with both sequence and expression data. The dataset was split into training (80%), validation (5%), and testing (15%) sets.

#### Model architecture

We developed a hybrid deep-learning model named GreyHound, which integrates sequence information and cell type–specific RBP expression to predict transcript stability. The model consists of three main components as follows:

1) Sequence module: A convolutional neural network (CNN) that processes transcript sequences. The sequences were one-hot encoded across four channels (A, C, G, and T) and passed through two convolutional blocks. Each block included a one-dimensional convolutional layer, leaky Rectified Linear Unit (ReLU) activation, batch normalization, max pooling, and dropout layers. Following these blocks, three residual parallel dilated convolutional layers with dilation rates of 1, 2, and 4 were applied to capture sequence features at different scales.

2) RBP expression module: A VAE pretrained on RBP expression data. The encoder component of the VAE compresses the 1378-dimensional RBP expression vector into a 50-dimensional latent space, capturing the essential patterns of RBP expression across cell types.

3) Integration module: A fully connected neural network that combines the flattened outputs from the sequence CNN and the latent representation from the RBP VAE to predict transcript stability.

#### VAE pretraining

First, a VAE was trained to encode the RBP expression profiles. The VAE consisted of an encoder with a 500-neuron hidden layer and a latent space of 50 dimensions. The loss function combined a reconstruction loss (mean squared error) and a Kullback-Leibler divergence term with a gradually increasing weight (β) to promote disentanglement of the latent space. The quality of the VAE was evaluated by calculating the Pearson correlation between the original expression values and the reconstructed values.

#### GreyHound model training

The encoder and µ (mean) components of the pretrained VAE were then transferred to the GreyHound model, with their weights initially frozen. The model was trained using mean squared error loss and the Adam optimizer with a learning rate of 0.001. Gradient clipping was applied to prevent exploding gradients.

Training proceeded in two stages as follows:

1) Initial training for 10 epochs with the VAE encoder weights frozen; and

2) Fine-tuning for an additional 5 epochs with all model parameters, including the VAE encoder, unfrozen

The model was trained using a batch size of 32. During training, the model’s performance was evaluated on the validation set after each epoch, and the model with the highest Pearson correlation coefficient on the validation set was selected as the final model. Every gene sequence–RBP profile pair constitutes an instance for model training.

#### Model evaluation

The performance of the model was evaluated on the held-out test set using Pearson correlation between predicted and observed RNA stability values. The model achieved a Pearson correlation coefficient of 0.624 on the test set, indicating a strong ability to predict transcript stability from sequence and RBP expression data. Ablation testing was performed to evaluate the generalizability of the model by leaving out entire groups of genes across all lines for testing (i.e., these genes are never seen by the model). We also tested leaving out each cell line and training the model on other lines before testing on the held-out line (LOO). In both cases, we observed only a slight reduction in the performance, highlighting that the model is robust to variations in train/val/test splits.

#### Interpretability analysis

To understand which sequence elements and RBPs contribute to stability predictions, we performed a saliency analysis. For each transcript, we calculated the gradient of the model’s output with respect to both the input sequence and RBP expression values.

For sequence interpretation, we identified regions with high gradient variance across cell lines, indicating sequences that differentially affect stability depending on cellular context. These salient regions were extracted and analyzed using FIRE to identify enriched motifs.

For RBP interpretation, we identified RBPs with high gradient values, suggesting their importance in determining variation in transcript stability. We then constructed a network connecting enriched motifs to RBPs that are likely to bind them on the basis of the correlation between RBP expression and the stability of transcripts containing specific motifs. This integrative approach allowed us to identify key sequence motifs and RBPs that modulate RNA stability in a cell state–specific manner, providing insights into posttranscriptional regulatory mechanisms in breast cancer cell lines.

### Clinical association studies

For survival analyses, we investigated the relationship between the expression of select RBPs (identified as important in our model) and patient outcomes in TCGA or METABRIC breast cancer datasets. Three types of survival end points were analyzed: disease-free survival (DFS), overall survival, and PFS.

For each survival analysis, we applied the following methodology as follows:

1) Patients with missing survival data were excluded from the analysis.

2) Survival times were capped at a maximum follow-up period (120 months for DFS and OS and 60 months for PFS) to minimize the effect of outliers and focusing on clinically relevant timeframes.

3) For each RBP of interest, we evaluated a range of expression thresholds to stratify patients into “high” and “low” expression groups. The threshold used for the Kaplan-Meier visualization was selected as the cut point that produced the strongest separation between survival curves [log-rank (Mantel-Cox) test], a standard approach for Kaplan-Meier plotting. This thresholding was used solely for visualization; all formal statistical associations were additionally evaluated using Cox proportional hazards models.

4) Kaplan-Meier curves were generated for the resulting patient groups, and differences were quantified using the log-rank (Mantel-Cox) test. Hazard ratios were calculated as the ratio of observed-to-expected events in the high-expression group divided by the same ratio in the low-expression group.

5) To adjust for potential confounding variables, Cox proportional hazards models were fitted with RBP expression status as the primary variable of interest, along with clinical covariates including tumor subtype, pathological stage, age, and prior diagnosis history.

In addition, we evaluated associations between RBP expression and clinicopathological features, such as tumor stage, using ANOVA followed by Tukey’s post hoc test for multiple comparisons.

### Cell culture

Human breast cancer cell lines MDA-MB-231 [MDA-parental, American Type Culture Collection (ATCC) HTB-26], MDA-LM2 (MDA-MB-231 highly metastatic derivative) (*24*), MDA-MB-453 (ATCC HTB-131), MCF7 (ATCC HTB-22), human embryonic kidney (HEK) 293T (293T, ATCC CRL-3216), and MDA-RBMS3-FLAG–tagged cells were all cultured in Dulbecco’s modified Eagle’s medium supplemented with 10% fetal bovine serum (FBS), penicillin (100 U/ml), streptomycin (100 μg/ml), and amphotericin B (1 μg/ml). The HCC1806 (ATCC CRL-2335), HCC38 (ATCC CRL-2314), and ZR-75-1 (ATCC CRL-1500) human breast cancer cell lines were cultured in RPMI 1640 medium supplemented with 10% FBS, penicillin (100 U/ml), streptomycin (100 μg/ml), and amphotericin B (1 μg/ml). Cell lines were routinely screened for *Mycoplasma* contamination with a PCR-based assay. All cell lines were grown in a humidified incubator at 37°C with 5% CO_2_.

### Knockdown cell lines

The shRNAs shRBMS3-1 (TRCN0000152525) and shRBMS3-2 (TRCN0000152012) were cloned into plasmid pLKO.1-Puro (Addgene, #8453). Three shRNA pLKO.1 plasmids each against RBM4B (TRCN0000158566, TRCN0000159492, and TRCN0000159895) and SAMDA4B (TRCN0000433715, TRCN0000412300, and TRCN0000005449) were purchased ready made from Millipore with the plasmid pLKO.1-Puro-Scramble (Addgene, #162011) acting as negative control. These plasmids were then cotransfected with lentiviral packaging plasmids pCMV-R8.91 (Addgene, #202687) and pMD2.G (Addgene, #12259) into HEK293T cells using the TransIT-Lenti Transfection Reagent (MirusBio, MIR 6600) according to the manufacturer’s instructions for lentivirus production. Virus was collected and filtered through a 0.45-μm filter, and 2 ml of viral media mixed with polybrene at final concentration 8 μg/ml (Sigma-Aldrich, TR-1003) was applied to transduce luciferase-labeled MDA-MB-231 and HCC1806 cell lines. The virus was removed after 8 hours, and cells were rested for 72 hours. For selection, puromycin was applied at 1 μg/ml until control cells (no virus) were all dead. Knockdown was assessed via qPCR.

For dual shRNA constructs, gene blocks of the dual shRNA cassettes containing either shScramble1-shScramble2, shScramble1-sh*TXNIP*, or sh*RBMS3*-1-sh*TXNIP* were ordered from TwistBio. The shRNAs used to generate these gene blocks were the following: sh*TXNIP* (TRCN0000262802), sh*RBMS3*-1 (TRCN0000152525), and two scramble sequences. These gene blocks were then cloned into the pLKO.1-Puro (Addgene, #8453) plasmid and then transduced as described above into luciferase labeled MDA-MB-231 cells. See table S1 for shRNA sequences.

### Overexpression cell lines

*RBMS3* overexpression cells were generated by stably transducing the pLX-304 lentiviral vector (Addgene, #25890) containing the RBMS3 open reading frame into MDA-LM2 cells.

### RBMS3 Flag-tagged MDA-MB-231

RBMS3 Flag-tagged cells were generated by stably transducing the lentiviral vector pLX302EF1a containing RBMS3 + N-ter 2xFLAG fusions into MDA-MB-231 breast cancer cells.

### RNA isolation and reverse transcription qPCR

Total RNA was isolated from cells using TRIzol Reagent (Invitrogen, 15596018) according to the manufacturer’s instructions. cDNA synthesis took place with Maxima H Minus Reverse Transcriptase (Thermo Fisher Scientific, EP0751) and for qPCR, PerfeCTa SYBR Green SuperMix (QuantaBio, 95056-500) was used with HPRT1 as endogenous control. See table S2 for qPCR primer sequences.

### Total RNA-seq

RNA was isolated from MDA-MB-231 cells with *RBMS3* knockdown and control in biological replicates using TRIzol reagent as described above. Libraries for RNA-seq were prepared using the SMARTer Stranded Total RNA-Seq Kit v3 - Pico Input Mammalian (Takara, 634485). Purified libraries were quantified on the TapeStation with D1000 HS tape (Agilent, 5067-5585). Sequencing was performed on Illumina NextSeq 500 with a PE75 run in Helen Diller Cancer Center. For the sh*RBM4B* and sh*SAMDA4B* knockdown cells lines and control, 200,000 cells each were collected in duplicate into 50 μl of DNA/RNA shield (Zymo) and sent to Plasmidsaurus for sequencing.

### RBMS3 CLIP-seq

#### Cell cross-linking

For cross-linking, MDA-MB-231 cells containing FLAG-tagged RBMS3 were harvested at ~90% confluency from 4- to 15-cm plates per replicate. On ice, the cells were rinsed with 1× PBS, and then in 1× PBS, the cells were irradiated using a 254-nm cross-linker set to 400 mJ/cm^2^ ultraviolet. Plates were placed back on ice and cells scraped, collected into tubes, spun down at 2000*g* at 4°C for 2 min, and then the remaining PBS was removed. Pellets were then frozen at −80°C.

#### Immunoprecipitation

Cell pellets were resuspended and lysed on ice for 10 min in 300 μl per plate of FLAG lysis buffer [25 mM tris (pH 7.5), 150 mM NaCl, 1% IGEPAL CA-630, and 5% glycerol] plus 3 μl of 100× protease inhibitor and 3 μl of ribonuclease (RNase) inhibitor. Deoxyribonuclease I was added at 30 μl per plate to lysate and incubated at 37°C for 10 min shaking at 1000 rpm. Lysate was then divided equally into two tubes. One tube is for 10 μl per plate of High RNase mix (RNase A 1:3000 + RNase I 1:100), and the other tube is for 10 μl per plate of Low RNase mix (RNase A 1:15,000 + RNase I 1:500). Lysate is then incubated at 37°C for 5 min. The two tubes are then combined and spun down at 4°C at ~20,000*g* for 20 min. FLAG Magnetic Agarose Beads (Pierce Anti-DYKDDDDK #A36797) that were prewashed twice in PBST (0.02% tween-20) and once in low-salt wash buffer (1× PBS with no Mg++ and no Ca++, 0.1% SDS, 0.5% sodium deoxycholate, and 0.5% IGEPAL CA-630) were then used for immunoprecipitation at 12.5 μl of beads per plate. Cleared supernatant was then added to beads and rotated end-over-end for 1 to 2 hours at 4°C. Beads were then washed twice with cold low-salt wash buffer, twice with cold high-salt wash buffer (5× PBS with no Mg++ and no Ca++, 0.1% SDS, 0.5% sodium deoxycholate, and 0.5% IGEPAL CA-630), and twice with cold PNK wash buffer [50 mM tris-HCl (pH 7.5), 10 mM MgCl2, and 0.5% IGEPAL CA-630].

#### Dephosphorylation

Immunoprecipitated RNP complexes were dephosphorylated on-beads by resuspending beads in 2.5 μl 10× PNK buffer [500 mM tris (pH 6.8), 5 mM MgCl_2_, and 50 mM dithiothreitol (DTT)], 2 μl T4 PNK (10 U/μl, NEB), 0.5 μl RNase inhibitor, 20 μl nuclease-free water. Samples were then incubated at 37°C for 20 min, shaking at 1350 rpm 15 s /5-min rest. Beads were then washed once with cold PNK wash buffer, once with cold high-salt wash buffer (>1 min) and twice with PNK wash buffer.

#### PolyA tailing RNP complexes

RNP complexes undergo polyA-tailing following dephosphorylation. On ice beads were resuspended in 0.8 μl of yeast PAP (Jena, 600 U/μl), 4 μl 5× yeast PAP buffer, 10 mM adenosine triphosphate (unlabeled), 0.5 μl of RNase inhibitor, and 13.7 μl of nuclease-free water. Samples were then incubated at 22°C for 5 min and with one 15-s shake at 1350 rpm. Beads were then washed twice with cold high-salt wash buffer and twice with cold PNK wash buffer.

#### N3-dUTP end labeling of RNP complexes

To N3–deoxyuridine triphosphate (dUTP) end-labeled RNA, the beads were resuspended in 0.4 μl of yeast PAP (Jena 600 U/μl), 2 μl of 5× yeast PAP buffer, 0.25 μl of RNase inhibitor, 2 μl of 10 mM N3-dUTP, and 5.35 μl of nuclease-free water. Samples were then incubated at 37°C for 20 min with shaking at 1350 rpm 15 s/5-min rest. Beads were then washed twice in cold high-salt wash buffer and twice in cold 1× PBS.

#### Dye labeling of N3-labeled RNP complexes

RNPs were then labeled by resuspending beads in 20 μl 800CW DBCO dye (0.2 mM in 1× PBS). Beads were incubated at 22°C for 30 min with shaking at 1350 rpm for 15-s/5-min rest. Beads were then washed once with cold high-salt wash buffer and once with cold PNK wash buffer. Then, beads were resuspended in loading buffer (1× NuPAGE loading buffer, 50 mM DTT, diluted in PNK wash buffer) and incubated at 75°C for 10 min shaking at 1000 rpm protected from light. Samples were then placed on magnet and eluate removed to clean tubes.

#### PAGE gel and transfer

Eluted samples were run on a 12-well Novex NuPAGE 4 to 12% bis-tris gel (1 mm thick) at 180 V in 1× MOPS running buffer at 4°C, protected from light. Samples were transferred to a Protran BA-85 nitrocellulose membrane in 1× NuPAGE transfer buffer with 10% ethanol at 30 V for 75 min. Membrane was imaged on a LI-COR Odyssey imaging instrument. The region of the membrane containing the RNA complexes was then cut out and placed in a clean tube.

#### Proteinase K digest and RNA capture

RNA was isolated from the membrane by digesting protein in 200 μl of protein K digestion buffer [100 mM tris-HCL (pH 7.5), 100 mM NaCl, 1 mM EDTA, and 0.2% SDS], and 12.5 μl of proteinase K at 55°C for 45 min shaking at 1100 rpm. To capture RNA, the supernatant was then transferred to a clean tube and adjusted to 0.5 M NaCl. This solution was then added to oligo d(T) magnetic beads prewashed twice with proteinase K buffer. Samples were then incubated at 25°C for 20 min shaking at 300 rpm with 1350 rpm increases for 10 s every 10 min. Beads were then washed twice with cold high-salt wash buffer and twice with cold 1× PBS. Samples are then eluted off of the beads in 8 μl of TE elution buffer [20 mM tris-HCl (pH 7.5) and 1 mM EDTA] and at 50°C for 5 min. Supernatant was transferred to PCR tubes in preparation for cDNA synthesis.

#### cDNA synthesis

For CLIP-seq library synthesis, the Takara SMARTer smRNA-Seq Kit was used with some modifications to the manufacturer’s protocol. Briefly, to the captured RNA from the previous step, 2.5 μl of smRNA Mix 1 and 10 μM unique molecular identifier (UMI) RT primer was added and incubated at 72°C for 3 min followed by 2 to 5 min on ice. Then, 6.5 μl of smRNA Mix 2, 0.5 μl of RNase inhibitor, and 2 μl of PrimeScript RT (200 U/μl) was added and samples incubated at 42°C for 60 min, 70°C for 10 min and then placed back on ice. See table S3 for primer sequences.

#### PCR amplification

To the 20 μl of cDNA from the previous step, 50 μl of 2× SeqAmp CB PCR buffer, 2 μl of SeqAmp DNA polymerase, 2 μl of 10 μM universal reverse primer, and 24 μl of nuclease-free water were added. Then, 2 μl of a unique 10 μM indexed TruSeq forward primer is added separately to each sample. Samples were amplified using the following thermocycler program: 98°C 1 min for 1 cycle, 98°C 10 s - 60°C 5 s - 68°C 10 s for 18 cycles, and hold at 4°C. Libraries were size selected using Zymo Select-a-Size MagBeads at 1.1× bead to PCR ratio according to the manufacturer’s instructions. Library size distribution and quantity were assessed using the Agilent D1000 ScreenTape System and an Agilent Tapestation 4200 according to the manufacturer’s instructions. Sequencing was performed on a HiSeq 4000 at the UCSF Center for Advanced Technologies. See table S3 for primer sequences.

#### CLIP-seq data analysis

Raw sequencing data from RBMS3-1 and RBMS3-2 samples were first processed by appending UMIs to reads using a custom Python script (append_umi.py). The processed reads were then analyzed using the CLIP Tool Kit (CTK) pipeline. The CTK pipeline included alignment to the human reference genome (hg38), filtering, and peak calling. CIMS (Cross-linking–induced mutation sites) analysis was performed to identify nucleotide deletions that represent direct protein-RNA cross-linking sites. These analyses resulted in 27,706 high-confidence RBMS3-binding sites (FDR < 10%, fold change >10) that were stored in the BED format file RBMS3_eclairCLIP.pool.tag.uniq.del.CIMS.fdr10.f10.bed.

To characterize the distribution of RBMS3-binding sites across genomic features, we performed systematic intersection of the binding sites with genomic annotations using BEDTools. The binding sites were categorized into 3’UTRs, 5’UTRs, coding sequences (CDS), introns, and intergenic regions by sequential subtraction:

1) 3’UTR peaks were directly intersected with the hg38 3’UTR annotation.

2) 5’UTR peaks were identified by excluding 3’UTR peaks and intersecting with 5’UTR annotations.

3) CDS peaks were identified by excluding 3′ and 5’UTR peaks and intersecting with exon annotations.

4) Intronic peaks were identified by excluding all previous categories and intersecting with intron annotations.

5) Intergenic peaks were calculated as the remaining sites.

We found that RBMS3 binding was predominantly enriched in 3’UTRs (73.3% of peaks), with smaller fractions in introns (10.9%), intergenic regions (12.1%), CDS (2.5%), and 5’UTRs (1.1%). This distribution was visualized using the UpSetR package in R, which illustrated the overlaps between binding sites in various genomic features.

To identify sequence motifs recognized by RBMS3, we extracted the sequences corresponding to the binding sites using twoBitToFa with the hg38 reference genome. The sequences were analyzed using FIRE, a mutual information-based motif discovery algorithm. Two approaches were used:

1) Nondiscovery mode: Using a previously defined RBMS3 motif pattern [AGT][AT]ATA[AT]A to validate its enrichment in our dataset.

2) Discovery mode: De novo identification of enriched sequence patterns.

FIRE analysis confirmed strong enrichment of A/U-rich motifs, with the most significant being the canonical AATAAA polyadenylation signal (*z* score = 1939.19). Additional enriched motifs included several variants of A/T-rich sequences, suggesting RBMS3’s preference for binding to such regions.

### Protein label-free quantification

In preparation for whole-protein analysis, *RBMS3* knockdown and control MDA-MB-231 cells were washed three times with PBS, scraped, pelleted, and snap frozen. After thawing stored pellets on ice, the samples were resuspended in lysis buffer (PreOmics) supplemented with complete mini protease inhibitor cocktail (20 mg/ml; Sigma-Aldrich) before preparation with iST96 kit (PreOmics). Cell lines were collected in biological triplicates, and samples were run in technical duplicates on the mass spectrometer.

A nanoElute system was attached in line to a timsTOF Pro mass spectrometer equipped with a CaptiveSpray source (Bruker). Chromatography was conducted at 40°C through a 25-cm reversed-phase C18 PepSep column (Bruker) at a constant flow rate of 0.5 μl/min. Mobile phase A was 98/2/0.1% Water/MeCN/Formic Acid (v/v/v) and phase B was MeCN with 0.1% formic acid (v/v). During a 108-min method, peptides were separated by a three-step linear gradient (5% to 30% B over 90 min, 30% to 35% B over 10 min, and 35 to 95% B over 4 min) followed by a 4-min isocratic flush at 95% for 4 min before washing and a return to low organic conditions. Experiments were run as data-dependent acquisitions with ion mobility activated in PASEF mode. Mass spectrometry (MS) and tandem MS spectra were collected with mass/charge ratio 100 to 1700 and ions with *z* = +1 were excluded.

Raw data files were searched using PEAKS Online Xpro 1.6 (Bioinformatics Solutions Inc.). The precursor mass error tolerance and fragment mass error tolerance were set to 20 parts per million and 0.03, respectively. The trypsin digest mode was set to semi-specific, and missed cleavages were set to 2. The human Swiss-Prot reviewed (canonical) database (downloaded from UniProt) and the common repository of adventitious proteins (downloaded from The Global Proteome Machine Organization) totaling 20,487 entries were used. Carbamidomethylation was selected as a fixed modification. Oxidation (M) was selected as a variable modification.

### Parallel reporter assays

#### Generation of dual reporter library

We performed a reporter assay by introducing the targets’ respective RBMS3-binding site sequences into the dual-promoter pBdLV-Puro-T2A-mCherry vector previously described (*10*). First, we selected RBMS3-binding site sequences of the listed targets based on the magnitude of ecCLIP peaks within the 3’UTR of the transcripts. For each binding site, we generated paired SCR control sequences that preserve the dinucleotide composition using ushuffle (*35*). Then, DNA oligonucleotides (116 base pairs long) were synthesized (IDT DNA) for these paired sequences and cloned into the pBdLV-Puro-T2A-mCherry reporter vector (*10*) through PacI digestion (NEB, R0547S) and HiFi assembly (NEB, E2621S) in an arrayed format. The plasmid library was pooled in an equimolar ratio, and lentivirus was prepared using TransIT-Lenti (MirusBio, MIR 6600) in HEK293T cells. Next, we transduced the viral library into MDA-MB-231 *RBMS3* knockdown and control cells for 8 hours, rested the cells, and sorted after 72 hours for mCherry-positive cells. For library preparation, we used the DNA/RNA co-extraction (Zymo Research, D7001), followed by reverse transcription of RNA samples with the following primer: 5’ CTCTTTCCCTACACGACGCTCTTCCGATCTNNNNNNNNNNNtggtctggatccaccggtccgg targeting GFP. Targeted amplification of genomic DNA (gDNA) and cDNA for mCherry (internal control for transcription rate) and GFP (indicative of mRNA stability) including the binding site sequences was performed with the primers listed below. Sequencing of amplified DNA and cDNA libraries was performed on HiSeq 4000 at the UCSF Center of Advanced Technologies. See table S4 for reporter target sequences.

#### Analysis of the parallel reporter assay data

We first collated sequence reads with identical sequences using the fastq2collapse.pl program (CTK tools) to remove PCR duplicates, generating collapsed FASTQ files. UMIs were extracted from these collapsed sequences using UMI tools. The adapter sequence (TGGTCTGGATCCACCGGTCCGGT) was then removed from the UMI-extracted reads using cutadapt (parameters: --trimmed-only -e 0.2 -g TGGTCTGGATCCACCGGTCCGGT -m 25), retaining only reads that contained the adapter and were at least 25 nucleotides long after trimming.

The processed RNA reads were then reverse-complemented using seqkit to align them with the reference sequences. For DNA libraries, a similar processing pipeline was used, but with a different adapter sequence (GTGGTCTGGATCCACCGGTCCGGTTTA), and a minimum length filter of 35 nucleotides.

A reference sequence file containing all candidate regulatory elements was indexed using BWA. Both RNA and DNA processed reads were aligned to this reference using BWA-MEM. Aligned reads were sorted and indexed using SAMtools. For RNA libraries, we performed UMI-based deduplication to account for PCR duplicates by collapsing reads with identical UMIs mapping to the same position using UMICollapse (parameters: -k 1 --algo adj --merge avgqual). Counts for each candidate regulatory element were obtained by extracting the reference sequence names from the alignment files and counting their occurrences using SAMtools and standard Unix tools.

We used DESeq2 for differential expression analysis. Size factors were calculated from the SCR sequences to normalize for sequencing depth differences. We performed two main comparisons:

1) For shCTRL samples, we tested the expression difference between reference (REF) and SCR sequences by calculating log_2_ fold changes and adjusted *P* values.

2) For shRBMS3 samples, we performed the same comparison between reference and SCR sequences.

The design formula for DESeq2 represented the combination of sequence type (REF/SCR) and library type (RNA/DNA). We used contrast lists to specifically test the interaction between sequence type and library type, effectively measuring the regulatory activity of the sequences while controlling for differences in DNA abundance. Statistical significance of the overall difference in regulatory activity between control and *RBMS3* knockdown conditions was assessed using a Wilcoxon signed-rank test.

### Animal studies

All animal studies were performed according to IACUC guidelines (IACUC approval number AN194337-01L). Metastatic lung colonization assays were done using age-matched female NSG mice (the Jackson Laboratory, 005557). In vivo bioluminescence was used to track metastasis and measured by injection of luciferin (Perkin-Elmer) followed by imaging on an IVIS instrument. Hematoxylin and eosin staining of lung tissue sections was used for histology.

### Dual-guide CRISPRi assays

#### Generation of dual guide library

The dual-guide CRISPR interference (CRISPRi) screen single guide RNA (sgRNA) library, containing 13 targets and 10 nontargeting controls, was designed according to a protocol previously described and oligos were synthesized (IDT). Briefly, two sgRNAs per target gene were inserted into the dual-guide plasmid pJR85 (Addgene, 140095) as the backbone with Golden-Gate assembly from pJR89 (Addgene, 140096) (*26*) using two-step cloning first with BstXI (NEB, R0113S) and BlpI (NEB, R0585S) and second with BsmBI-v2 (NEB, R0739S). The sgRNA sequences were selected from (*36*). See table S5 for sgRNA target sequences.

The two-step cloning was performed using an arrayed format, and the resulting plasmids were pooled at an equimolar ratio. The dual sgRNA plasmid library was then transduced into Zim3-CRISPRi (*37*)–ready MDA-MB-231 cells via lentiviral transduction as described above. The cells were then plated (in vitro arm) and injected into tail veins of NSG mice (in vivo arm). For the in vitro arm, 1 × 10^5^ cells were plated and cells from day 0 as well as cells after 10 doublings had occurred were collected for library preparation. For the in vivo arm, 2.5 × 10^5^ cells were injected via tail vein into NSG mice and lungs were collected at ~5 weeks. The gDNA from both experimental arms was isolated, and library preparation was done according to a protocol previously described in (*26*). Sequencing was performed on NovaSeq X 1.5B with a PE100 run.

#### Analysis of the CRISPRi assay data

Sequencing data from in vitro and in vivo CRISPR screens was processed using a multistep computational workflow. Raw FASTQ files were first trimmed to remove adapter sequences using Cutadapt (v4.9). For each sample, forward reads were trimmed for the 5′ adapter sequence (GTTTCAGAGCTAAGCACAAGAGTGCATAGCAA) and reverse reads for the 3′ adapter sequence (CCATGTTTCTGGCTTTCCACAAGATATATAAA). The trimming parameters were set to retain only reads with a fixed length of 19 nucleotides to ensure precise sgRNA sequence capture. Paired-end reads were processed together. The trimmed reads were aligned to a custom reference containing dual-guide sequences using Bowtie2 (v2.3.4.2). The alignment was performed with sensitive parameters and end-to-end mode (--sensitive --end-to-end -N 1) to ensure accurate mapping of sgRNA sequences. Only properly paired reads that aligned to the reference were retained using SAMtools filtering parameters (-f 0x1 -f 0x2 -F 0x4 -F 0x8), generating BAM files for each sample.

Read counts for each sgRNA were extracted from the aligned BAM files using SAMtools. For each sample, the third field (RNAME) in the SAM format was extracted to identify the target sgRNA, and the occurrences were counted using the Unix commands sort and uniq -c. The resulting count tables were stored in tab-delimited format. The count data were processed in R, where individual count files were merged into a combined count matrix. To account for paired-end reads, the raw counts were divided by two to avoid double-counting. The matrix was structured with sgRNAs as rows and samples as columns, with nine total samples representing as follows:

1) Three day 0 replicates (RBMS3-TL-D0-1, RBMS3-TL-D0-2, and RBMS3-TL-D0-3);

2) three day 10 in vitro samples (RBMS3-TL-D10-1, RBMS3-TL-D10-2, and RBMS3-TL-D10-3); and

3) three in vivo tumor samples (RBMS3-TL-M-1, RBMS3-TL-M-2, and RBMS3-TL-M-3)

Differential abundance analysis was performed using the DESeq2 package (v1.42.0) in R to identify sgRNAs with significant changes in abundance between conditions. Nontargeting control sgRNAs (prefixed with “NT_”) were used for normalization and as a reference for calculating size factors to account for sequencing depth differences between samples.

The experimental design included three conditions (D0, D10, and TL-M) with three biological replicates each. Three key comparisons were analyzed:

1) In vivo (TL-M) versus day 0 (D0)

2) In vitro (D10) versus day 0 (D0)

3) In vivo (TL-M) versus in vitro (D10)

Samples were grouped by condition, with day 0 samples serving as the reference for both in vitro and in vivo comparisons. The DESeq2 analysis pipeline included estimating size factors, calculating dispersions, and fitting negative binomial models to determine log_2_ fold changes and adjusted *P* values for each sgRNA across conditions.

A scatter plot was generated to compare the log_2_ fold changes of sgRNAs in the in vivo screen (*x* axis) versus the in vitro screen (*y* axis), both relative to day 0. Each point represents an individual sgRNA, with color indicating the statistical significance (adjusted *P* value). Significantly depleted or enriched sgRNAs (adjusted *P* value <0.01) were labeled on the plot. This visualization allowed for direct comparison of sgRNA behavior in the two screening conditions and identification of context-specific essential genes.

### Proliferation assays

On day 0, 5 × 10^4^ cells per well were seeded onto six-well plates in biological triplicates. Cells were then trypsinized, collected, and stained with 0.4% Trypan Blue Solution (Gibco) to determine cell viability on days 3 and 5. The number of viable cells was counted using a TC20 Automated Cell Counter (Bio-Rad). A linearized exponential growth model [$\text{ln}\left(N_{t}\right)=\text{ln}\left(N_{0}\right)+r\times t$, with time t (days), proliferation rate r (day^−1^) and number of cells N] was used to fit a proliferation rate for each cell line. To compare proliferation rates and test for significant differences, an unpaired two-sided *t* test was used.

### Invasion assays

For invasion assays, *RBMS3* knockdown and control MDA-MB-231 cells were seeded into Corning BioCoat Matrigel Invasion Chambers with 8.0-μm polyethylene terephthalate membranes (Corning) at a final concentration of 8 × 10^4^ cells per chamber in 500 μl of serum-free medium. Then, 750 μl of complete culture medium containing 10% FBS, as a chemoattractant, was added to the lower chamber. The cells were incubated at 37°C for 24 hours. After incubation, the inserts were gently washed three times with cold PBS, and the cells were fixed using 100% methanol. Nonmigrated cells on the top of the inserts were removed using cotton swabs. Following fixation, the cells that had migrated to the bottom of the inserts were washed again with PBS and stained with 0.4% crystal violet. Migrated cells were counted from three random fields per insert at a magnification of 20×, and the results were averaged from at least three biological replicates.

### Measurements in clinical samples

We measured *TXNIP* expression using qPCR in 96 clinical samples across all stages of breast cancer, namely 5 normal epithelial, 23 stage I, 30 stage II, 29 stage III, and 9 stage IV metastatic biopsies (Origene, BCRT102, BCRT103), from which 90 samples yielded sufficient amount of cDNA. HPRT1 was used as an endogenous control and relative *TXNIP* expression levels were measured using the primers listed in table S6. See table S6 for *TXNIP* qPCR primer sequences.
