# Supplementary material for: Integrative analysis of mRNA stability regulation uncovers a metastasis-suppressive program in breast cancer
Source: Sci Adv. 2026 Mar 11;12(11):eaea9061. doi: 10.1126/sciadv.aea9061 (PMC12978252; doi:10.1126/sciadv.aea9061)
Supplement: Supplementary file 1 — Figs. S1 to S6 Tables S1 to S6 [file sciadv.aea9061_sm.pdf]

Science Advances
AAAS

Supplementary Materials for

Integrative analysis of mRNA stability regulation uncovers a metastasis-suppressive program in breast cancer

Heather Karner *et al.*

Corresponding author: Hani Goodarzi, hani@arcinstitute.org

Sci. Adv. **12**, eaea9061 (2026)
DOI: 10.1126/sciadv.aea9061

This PDF file includes:

Figs. S1 to S6
Tables S1 to S6

Figure S1

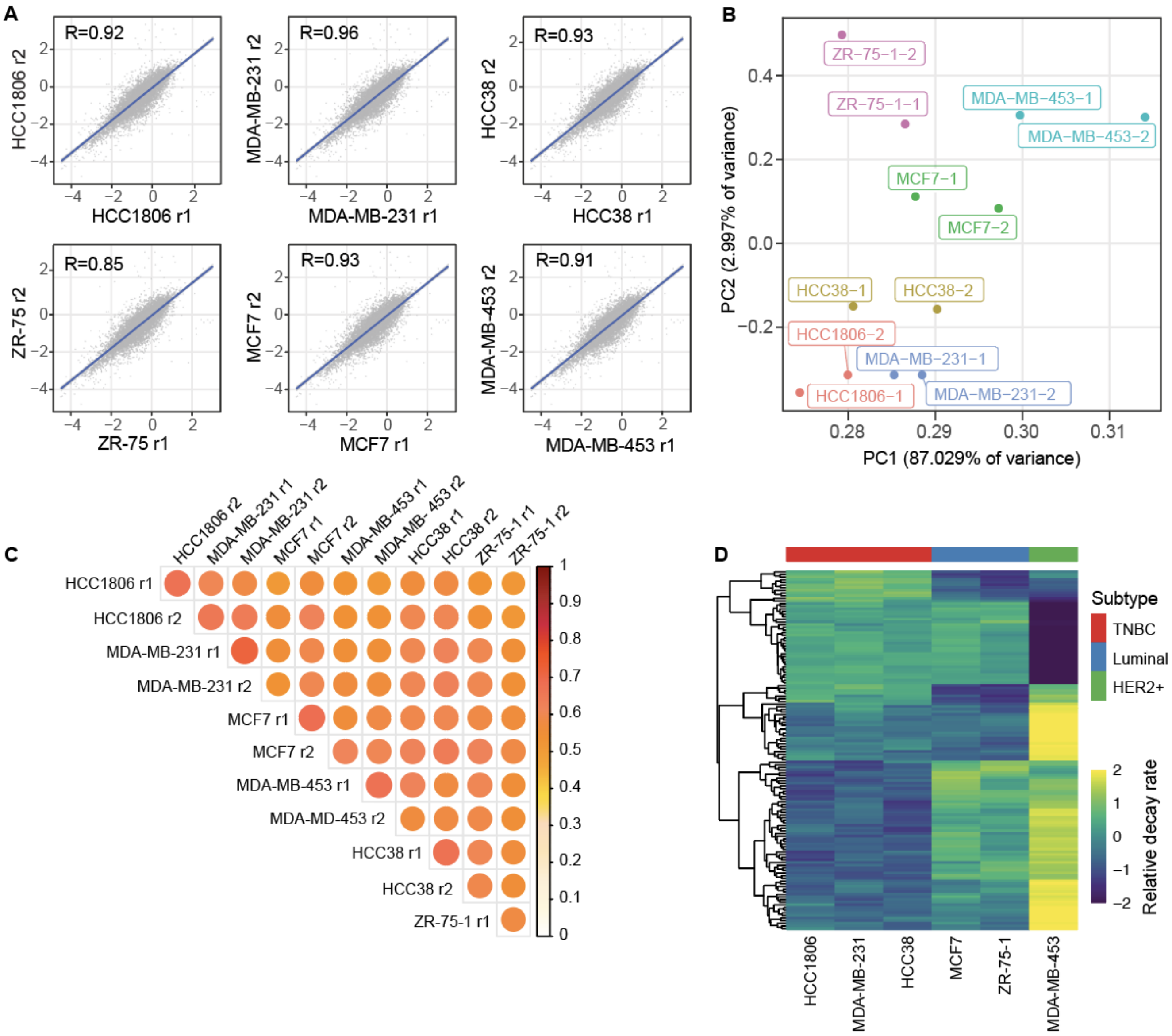

Figure S1: SLAM-based mRNA decay estimates across biological replicates, cell lines, and breast cancer subtypes. (**A**) Scatter plots demonstrating the Pearson correlation between mRNA decay rate estimates between biological replicates for each cell line. (**B**) PCA plots summarizing the mRNA decay rate measurements across samples. (**C**) Pairwise Pearson correlation coefficients for RNA decay rate measurements across all samples and replicates. Circle size and color intensity reflect correlation strength (scale: 0 to 1). (**D**) A heatmap of normalized mRNA decay rate estimates across cell lines for 136 subtype-specific genes. Normalization of decay rates using z-scores per row.

Figure S2

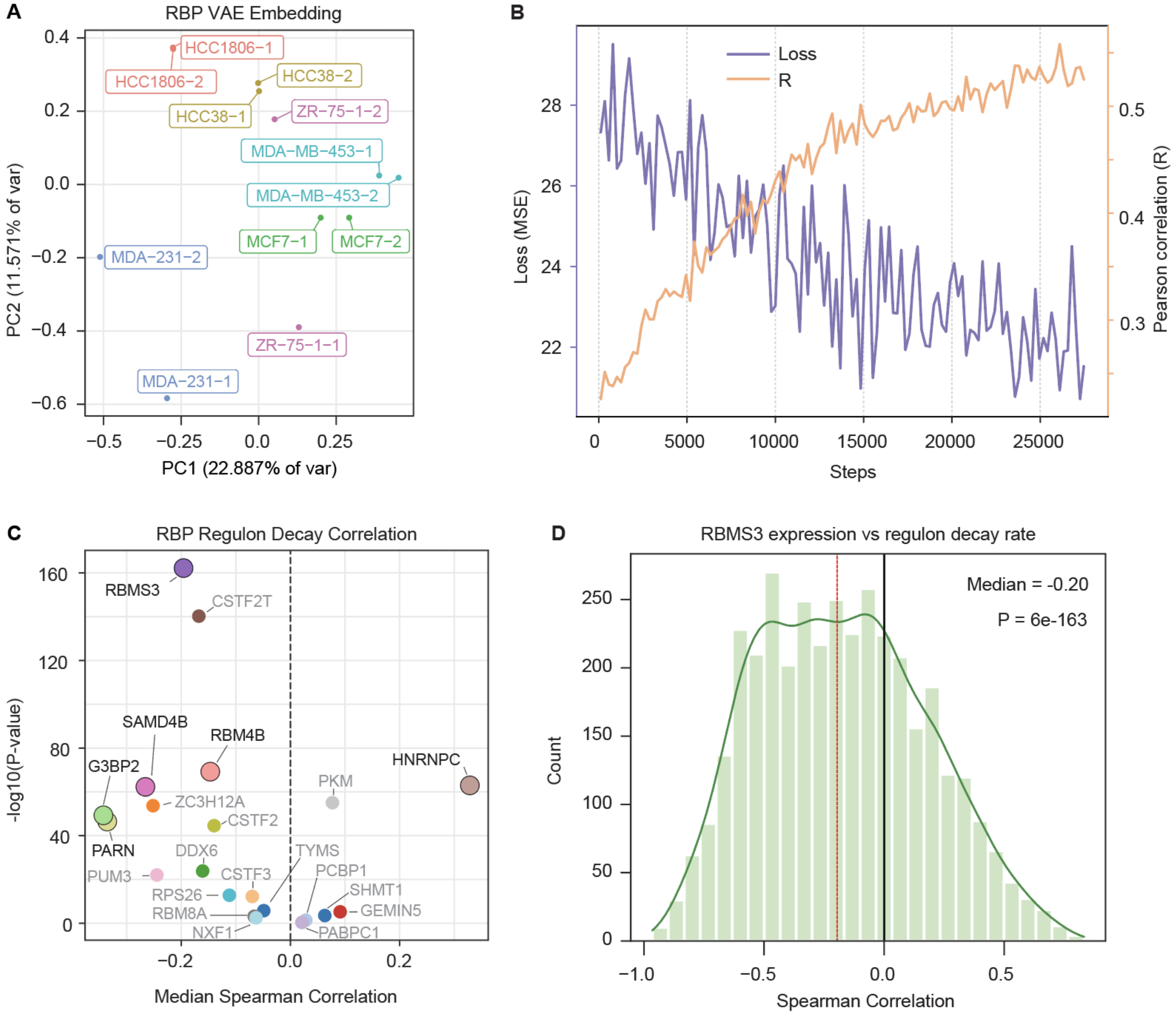

Figure S2: Model training and nomination of RBMS3 as a regulator of differential mRNA stability across breast cancer cell lines. (**A**) PCA plot of the embedding learned for each sample using the expression of RBPs. This is to highlight that RBP expression can largely capture cell states. (**B**) The training loss and Pearson correlation curves for the model. The model was stopped after 15 epochs (approximately 2000 steps per epoch) when the validation R had largely plateaued. (**C**) Volcano plot of RBP motif-regulon decay correlations across breast cancer cell lines. (**D**) The distribution of Spearman correlation coefficient between RBMS3 expression and decay rates for the respective putative target transcripts. The median and associated *p*-value (Wilcoxon test) are also shown.

Figure S3

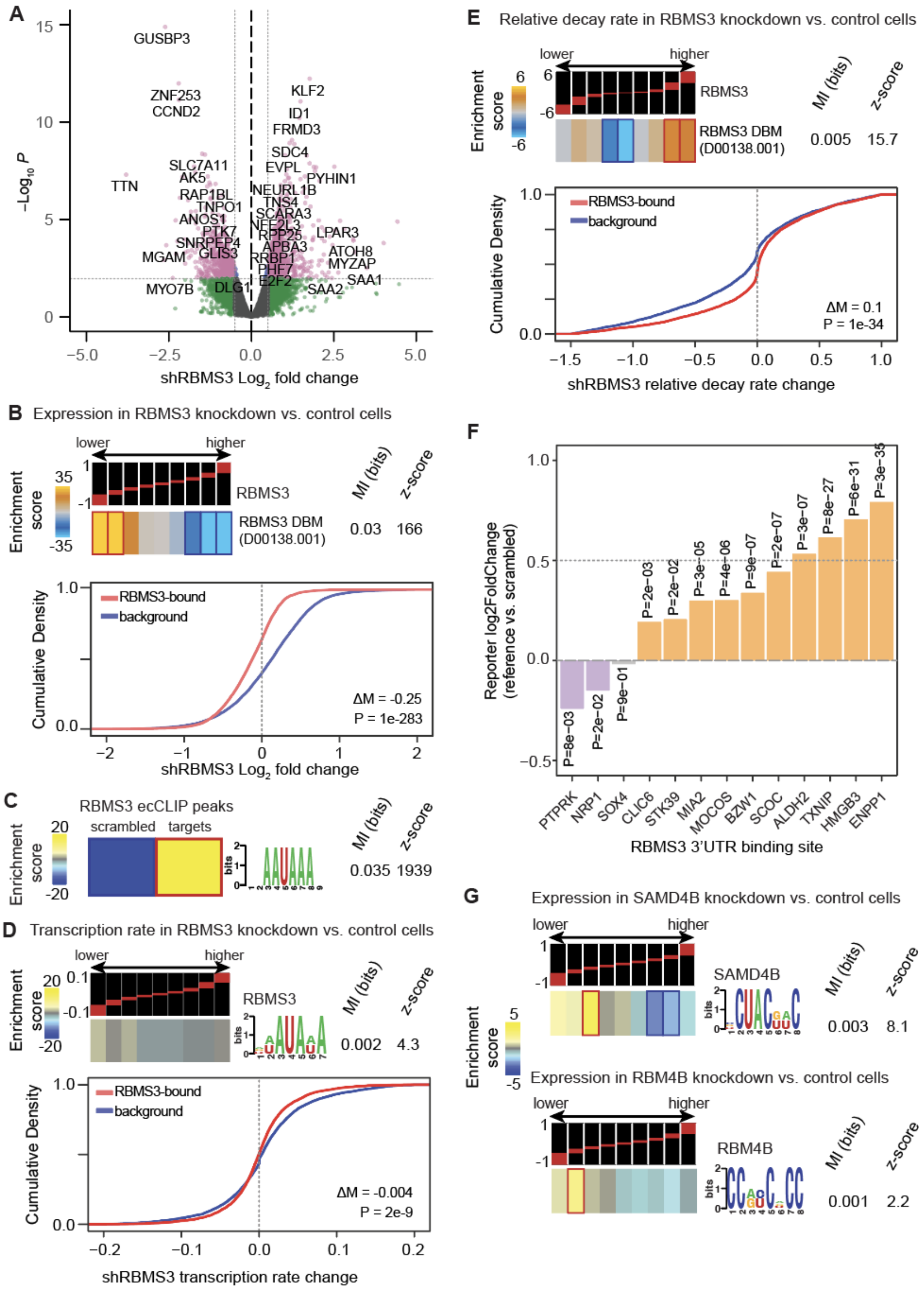

Figure S3: RBMS3 expression impacts putative and experimental target regulon expression and stability. (**A**) Volcano plot of differential gene expression in MDA-MB-231 RBMS3 knockdown cells (N=2 biological replicates) versus control cells (N=2 biological replicates). (**B**) Enrichment and depletion patterns of the DeepBind RBMS3 regulon shows RBMS3 target gene enrichment among the genes that are downregulated

in RBMS3 knockdown cells. (**C**) De novo motif discovery in RBMS3 binding sites, with dinucleotide-invariant scrambled sequences as background, identified an AUA motif, similar to the one we identified based on our analysis of GreyHound's feature importance scores. (**D**) Enrichment and depletion patterns of the transcription rates for transcripts containing the AUA sequence element in MDA-MB-231 RBMS3 knockdown versus control cells. (**E**) Enrichment and depletion patterns of the relative decay rates of DeepBind RBMS3 regulon in MDA-MB-231 RBMS3 knockdown versus control cells. (B, D-E, G) Shown are enrichment patterns along with calculated mutual information (MI) and associated Z-score (see (*13*) for details), in addition to the cumulative density with delta median and Mann-Whitney U test. (**F**) The contribution of each cloned target binding sequence on GFP expression by comparing their relative abundances in GFP mRNA to that of their scrambled controls. We performed this analysis in both control and RBMS3 knockdown cells, and used a paired Wilcoxon signed rank test to report a *p*-value. (**G**) Enrichment and depletion patterns of SAMD4B and RBM4B target motif–containing transcripts relative to RNA stability changes in MDA-MB-231 cells with knockdown of SAMD4B and RBM4B respectively versus control cells.

Figure S4

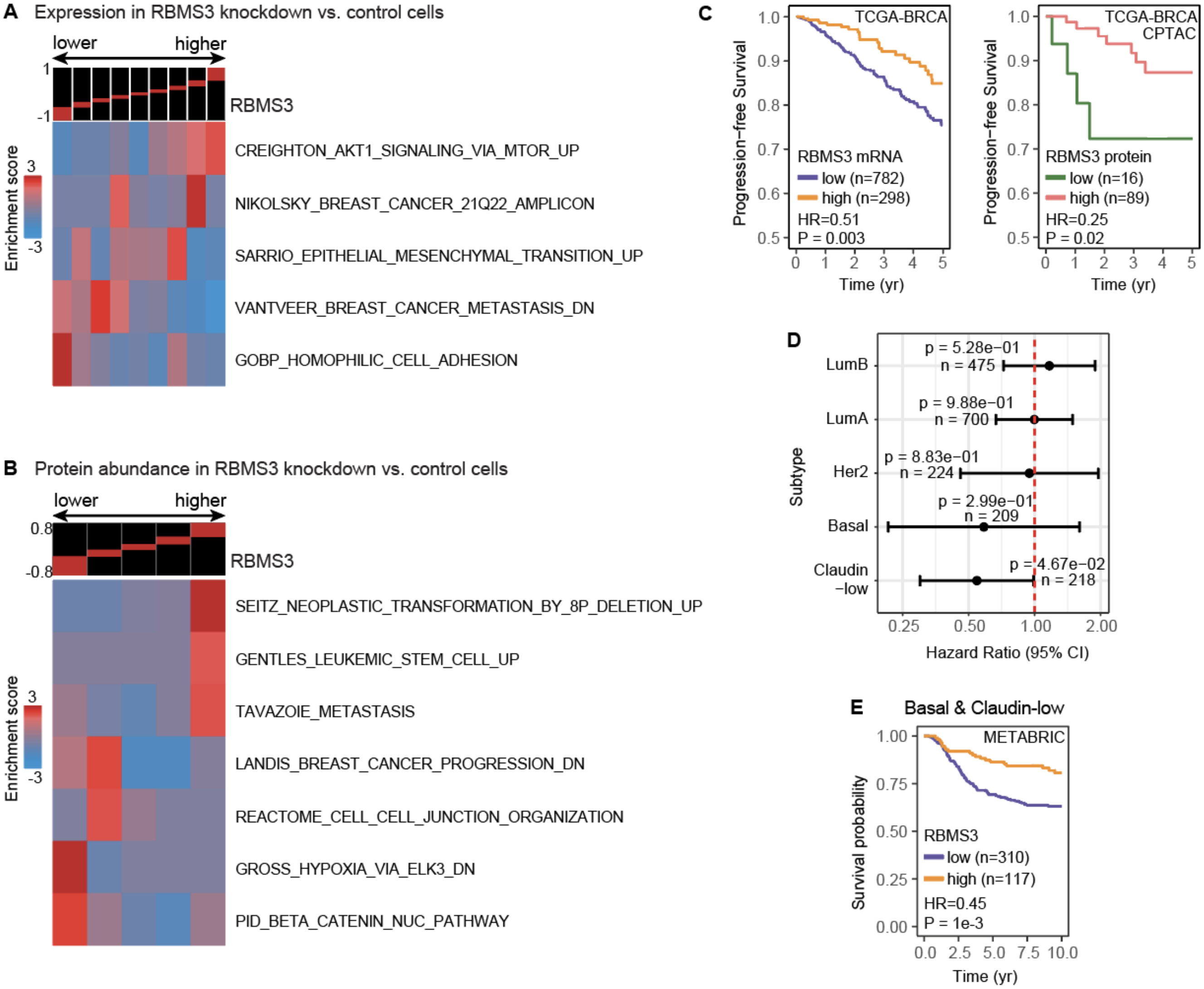

Figure S4: RBMS3 is a predictor of breast cancer progression. (**A**) Cancer pathway associated gene expression enrichment analysis in RBMS3 knockdown versus control cells. (**B**) Cancer pathway associated protein abundance enrichment analysis in RBMS3 knockdown versus control cells. (**C**) Analysis of progression-free survival in the TCGA-BRCA and TCGA-BRCA CPTAC cohorts relative to RBMS3 expression. TCGA-BRCA (The Cancer Genome Atlas – Breast Invasive Carcinoma Cohort) and TCGA-BRCA-CPTAC (Clinical Proteomic Tumor Analysis Consortium) data sets were downloaded from the genome data commons. Hazard ratios estimated using Cox proportional hazards models. Statistical comparison was done using Mantel-Cox test. (**D**) Hazards ratio 95% confidence interval of subtypes in the METABRIC cohort (*21*). Likelihood ratio test was used to assess significance. (**E**) Analysis of survival probability in the Basal and Claudin-low subtypes of the METABRIC cohort (*21*). Hazard ratios estimated using Cox proportional hazards models. Statistical comparison was done using Mantel-Cox test.

Figure S5

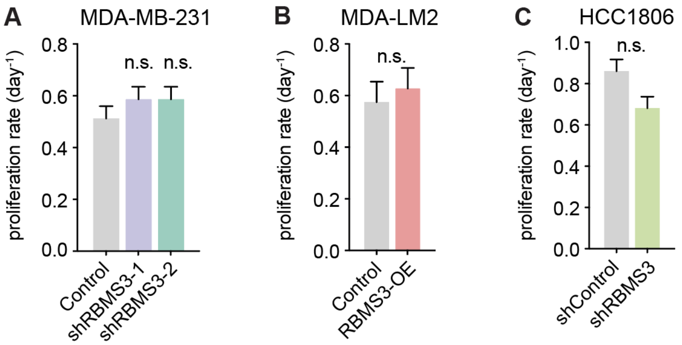

Figure S5: RBMS3 expression does not affect proliferation. (**A**) *In vitro* proliferation rate in RBMS3 knockdown and control cells (two independent hairpins in MDA-MB-231 cells; N = 3 biological replicates). (**B**) *In vitro* proliferation rate in RBMS3 overexpression and control cells (in MDA-MB-231 cells; N = 3 biological replicates). (**C**) *In vitro* proliferation rate in RBMS3 knockdown and control cells (best performing hairpin was used in HCC1806 cells; N = 3 biological replicates). (A-C) The t-statistic from a linear model was used to assess significance.

Figure S6

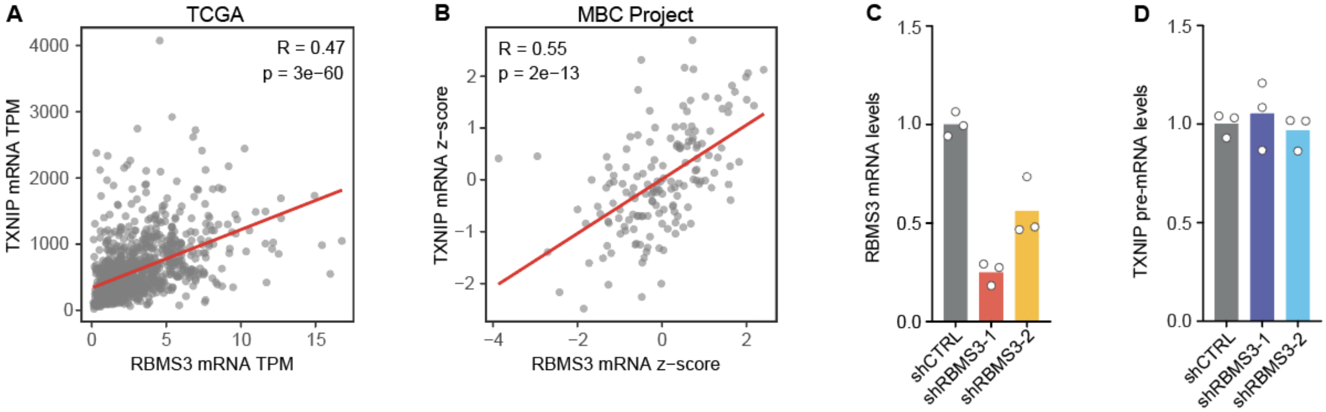

Figure S6: RBMS3 modulates TXNIP expression in a post-transcriptional manner. (**A-B**) Comparative analysis of TXNIP to RBMS3 expression in (A) TCGA and (B) MBCProject datasets using Pearson correlation with t-statistic for non-zero correlation. (**C**) Expression levels of RBMS3 as measured by qPCR in RBMS3 knockdown and control cells. Sufficient knockdown of RBMS3 is seen. (**D**) Expression levels of TXNIP pre-mRNA in RBMS3 knockdown and control cells as measured by qPCR.

shRNA	Sequence
shRBMS3-1	5' - GACATCTATCACGCCATTCAT
shRBMS3-2	5' - CACAAATCAGTGCAAAGGTTA
shTXNIP	5' - ATCAGTCAGAGGCAATCATAT
Scramble 1	5' - CCGCAGGTATGCACGCGT
Scramble 2	5' - GACATCTATCACGCCATTCAT
shRBM4B-1	5' - CTCAACTCTACTTCTGTTGAT
shRBM4B-2	5' - CCTTTCACTCTGTTCCTTATA
shRBM4B-3	5' - GCTTCATGTTTCAGTAAACAA
shSAMD4B-1	5' - GCTCAGAAACATTTGACATTT
shSAMD4B-2	5' - GTGGGCTGGCTCAGCTATATA
shSAMD4B-3	5' - GTGGGCTGGCTCAGCTATATA

Table 1: shRNAs for generation of knockdown cell lines.

qPCR Primer	Sequence
RBMS3-F	5' - CACCACTGACCAGGACCTAA
RBMS3-R	5’ - CCTTGAGAGATGCTACCGCT
TXNIP-F	5’ - GAATACATGTTCCCGAATTGTGG
TXNIP-R	5’ - ACTTCTGAGTCAGCACCTTG
HPRT1-F (Control)	5' - TGCTGAGGATTTGGAAAGGG
HPRT1-R (Control)	5' - ACAGAGGGCTACAATGTGATG

Table 2: RBMS3 and TXNIP primers for quantitative PCR to evaluate knockdown efficiency.

RT/PCR Primers	Sequence
UMI RT Primer	5' - CAAGCAGAAGACGGCATACGAGATNNNNNNNNGTGACTGGAGTTCAGACGTGTGCTCTTCCGATCTTTTTTTTTTTTTTT
Universal Forward Primer	5' - AATGATACGGCGACCACC
Universal Reverse Primer	5' - CAAGCAGAAGACGGCATACGAG
**TruSeq Primer**	
**I5 Index Number**	**Barcode Sequence**
1	5' - TCATTAGT
2	5' - ATAGAGGC

Table 3: Primers for RBMS3 CLIP-seq library generation.

Target	Sequence
ALDH2_wt	aaacgcgtgctacgttacattaaggcaactgcTACACCCTGCTTTGTATTCTGGGCTAAGATTCATTAAAAACTAGCTGCTCTTAACttactgggtataaaccggaccggtggatc
ALDH2_scr	aaacgcgtgctacgttacattaaggcaactgcTAAAGCTAGCTCTTACTCAAATTCCTAAACCTGCTGTTATGGGACATTTTACTGCttactgggtataaaccggaccggtggatc
BZW1_wt	aaacgcgtgctacgttagccaaaagtttgtcatttaaaagttcattttgagggaataacaTGTAATATAATTTGAAATAAAggtatagtaaccttaataaaccggaccggtggatc
BZW1_scr	aaacgcgtgctacgttagccaaaagtttgtcatttaaaagttcattttgagggaataacaTGATTGTAAAATAATTATAAAggtatagtaaccttaataaaccggaccggtggatc
CLIC6_wt	aaacgcgtgctacgttattgtattcttagacattaaaatgattgacataaacTCTTTGTGCCTTGAAAATGAAacaaattataaaaatgtttaaatgtaaaccggaccggtggatc
CLIC6_scr	aaacgcgtgctacgttattgtattcttagacattaaaatgattgacataaacTTGCCTCTTGTGAAAAATTGAacaaattataaaaatgtttaaatgtaaaccggaccggtggatc
ENPP1_wt	aaacgcgtgctacgttacaactttgagggacgatctTTGAATATACTTACCTATTATAAAATCTTACTTTGTATTTGTATTTAAaaaagaaaaatattaaaccggaccggtggatc
ENPP1_scr	aaacgcgtgctacgttacaactttgagggacgatctTTTTATATATTATAAAATGTACTGTTTCTTTACCTATAATTAACTTGAaaaagaaaaatattaaaccggaccggtggatc
HMGB3_wt	aaacgcgtgctacgttaacacgattcgcaaCGTTCTGTTATTTTTTTTGTATGTTTAGAATGCTGAAATGTTTTTGAAGTTaaataaacagtattactaaaccggaccggtggatc
HMGB3_scr	aaacgcgtgctacgttaacacgattcgcaaCTGTGCGTGTTATTAATTGAATTTTTTTTGAATGTGAGTCTTTTTTAAGTTaaataaacagtattactaaaccggaccggtggatc
MIA2_wt	aaacgcgtgctacgttatgtatttactgtggttgtggacaaatgTGAAAGTAACTTTATGCTTAAATAAATTATAGTTGATTTAAAGatttgtttggtaaaccggaccggtggatc
MIA2_scr	aaacgcgtgctacgttatgtatttactgtggttgtggacaaatgTAGTACTTTTAATTAATAAAGAGCTATTGTAAAAATTTGAATGatttgtttggtaaaccggaccggtggatc
MOCOS_wt	aaacgcgtgctacgttatgagtgttggttgtattattctttattttttgtatgacttATGATATTCATGATAATTAAAACGTTTTGGAACAAttcataaaccggaccggtggatc
MOCOS_scr	aaacgcgtgctacgttatgagtgttggttgtattattctttattttttgtatgacttATGGTGATTTTTATGAATTACATAAAATCAAACGAttcataaaccggaccggtggatc
NRP1_wt	aaacgcgtgctacgttatgtaaaataACATATTTTTTCTTTATGGAAATCTATAAAACTTTCTGTAGTAAAATGTTTTCATTTTACTGgtatattattaaaccggaccggtggatc
NRP1_scr	aaacgcgtgctacgttatgtaaaataATGGTAAATTTAAATTTCTTAGTTCTAACTTCTTAAATTTTGTGACTTTCATTAACATATTGgtatattattaaaccggaccggtggatc

PTPRK_wt	aaacgcgtgctacgttattctagattgccagctcatgacatggtgctTATAAAGATTTAATTAAAGTAAGAATGAAATAAAGTTTTTATAATTATAataaaccggaccggtggatc
PTPRK_scr	aaacgcgtgctacgttattctagattgccagctcatgacatggtgctTGAATTAGATTATTATATAGAAAAAAAAAAGTTTTATAATATTAGTTAAataaaccggaccggtggatc
SCOC_wt	aaacgcgtgctacgttattttgtgacctaaTTTACAGACATTTAAATTGTGTTGCAGTTCTGCTTTGCCGTTTAATAAAAAGCTATTTCAGAGGTTgtaaaccggaccggtggatc
SCOC_scr	aaacgcgtgctacgttattttgtgacctaaTTAAAAGGTCAATTTTAGTTGTTGATAGTGTGAGCGCACTTTTTAGCCTTTGCAAATTAACTTCATgtaaaccggaccggtggatc
SOX4_wt	aaacgcgtgctacgttattgccatccatcctgtgcaatatgccGTGTAGAATATTTGTCTTAAAattcaaggccacaaaaacaatgtttgggggaaataaaccggaccggtggatc
SOX4_scr	aaacgcgtgctacgttattgccatccatcctgtgcaatatgccGTTTGTATTAAATCTGTAGAAattcaaggccacaaaaacaatgtttgggggaaataaaccggaccggtggatc
STK39_wt	aaacgcgtgctacgttaccattctatTGTTTACACAACGATTACTCGAAGATGACTGCAAAGGTAAAAGGAAAATAAAAGTGTATTGCACAATGAgtaaaccggaccggtggatc
STK39_scr	aaacgcgtgctacgttaccattctatTGAATACAACACGCGGATGTTAGTAATGTTGAGAAAACAAGCATGGTTAATAAACTCTTGAAGAAACAAgtaaaccggaccggtggatc
TXNIP_wt	aaacgcgtgctacgttacttaaccagatatatttttaccccagatggGGATATTCTTTGTAAAAAATGAAAATAAAGTTTTTTTAATGGAAAAAaaataaaccggaccggtggatc
TXNIP_scr	aaacgcgtgctacgttacttaaccagatatatttttaccccagatggGGTTTGGAATATTTGAAAAATTTAAAATAAATTGTTTCTAAGAAAAAaaataaaccggaccggtggatc

Table 4: Reporter sequences for RBMS3 binding sites and scrambled control.

sgRNA target	Sequence
HK_HMGB3	ATTTTGCCCCTGGTTCTTCCACCTTGTTGGGGCCGCAGCCGGGAACCCAGTTTCAGAG<br>CGAGACGTGCCTGCAGGATACGTCTCAGAAACATGGCGGGAACCCAGGGCCCCCGGT<br>TTAAGAGCTAAGCTGCCAGTTCATTTCTTAGGG
HK_NRP1	ATTTTGCCCCTGGTTCTTCCACCTTGTTGGAGACACCCGGACCTCCCCTGTTTCAGAGC<br>GAGACGTGCCTGCAGGATACGTCTCAGAAACATGGTGGCGCCCAGGGGAGGTCCGTT<br>TAAGAGCTAAGCTGCCAGTTCATTTCTTAGGG
HK_CLIC6	ATTTTGCCCCTGGTTCTTCCACCTTGTTGGGCCGCGGAGGTGCAGTGCAGTTTCAGAG<br>CGAGACGTGCCTGCAGGATACGTCTCAGAAACATGGGCGGGCCGGGCACTTCCAAGT<br>TTAAGAGCTAAGCTGCCAGTTCATTTCTTAGGG
HK_ALDH2	ATTTTGCCCCTGGTTCTTCCACCTTGTTGGAGGCGGGGCCCGAAGCGGGGTTTCAGAG<br>CGAGACGTGCCTGCAGGATACGTCTCAGAAACATGGGCAGCGCGCAACATCGCAGGT<br>TTAAGAGCTAAGCTGCCAGTTCATTTCTTAGGG
HK_SCOC	ATTTTGCCCCTGGTTCTTCCACCTTGTTGGTGAGGCGCCGGGAGAAGGAGTTTCAGAG<br>CGAGACGTGCCTGCAGGATACGTCTCAGAAACATGGGTGTCCCGGCCTGAGGTGTGT<br>TTAAGAGCTAAGCTGCCAGTTCATTTCTTAGGG
HK_ENPP1	ATTTTGCCCCTGGTTCTTCCACCTTGTTGGGGGCCACGATGGAGCGCGAGTTTCAGAG<br>CGAGACGTGCCTGCAGGATACGTCTCAGAAACATGGGAGCGCGACGGCTGCGCGGGT<br>TTAAGAGCTAAGCTGCCAGTTCATTTCTTAGGG
HK_BZW1	ATTTTGCCCCTGGTTCTTCCACCTTGTTGGCCCGATGTACCGGCAACTGGTTTCAGAGC<br>GAGACGTGCCTGCAGGATACGTCTCAGAAACATGGGCGTTAGTTCCGGTCGCAGGTTT<br>AAGAGCTAAGCTGCCAGTTCATTTCTTAGGG
HK_TXNIP	ATTTTGCCCCTGGTTCTTCCACCTTGTTGGCTCCTTTGGAGAAAAAGAGGTTTCAGAGC<br>GAGACGTGCCTGCAGGATACGTCTCAGAAACATGGATCTCCACAAGCACTCCTTGTTTA<br>AGAGCTAAGCTGCCAGTTCATTTCTTAGGG
HK_SOX4	ATTTTGCCCCTGGTTCTTCCACCTTGTTGGGGCCGCGAGAAACTTGCATGTTTCAGAGC<br>GAGACGTGCCTGCAGGATACGTCTCAGAAACATGGGAGGCGCGAGGCGGAATTGGTT<br>TAAGAGCTAAGCTGCCAGTTCATTTCTTAGGG
HK_MOCOS	ATTTTGCCCCTGGTTCTTCCACCTTGTTGGCCGGGACCCGAGCGCACCGGTTTCAGAG<br>CGAGACGTGCCTGCAGGATACGTCTCAGAAACATGGAGGTAGCGCCGCTCTCACTGTT<br>TAAGAGCTAAGCTGCCAGTTCATTTCTTAGGG
HK_STK39	ATTTTGCCCCTGGTTCTTCCACCTTGTTGGCGCCGAAGCCAGCTAGGAGGTTTCAGAG<br>CGAGACGTGCCTGCAGGATACGTCTCAGAAACATGGGCCGGCCGAGAGGTGTCGGGT<br>TTAAGAGCTAAGCTGCCAGTTCATTTCTTAGGG
HK_PTPRK	ATTTTGCCCCTGGTTCTTCCACCTTGTTGGTGATAGATGGCGAATGGAAGTTTCAGAGC<br>GAGACGTGCCTGCAGGATACGTCTCAGAAACATGGCCCTGGCCAGGATCATGAAGTTT<br>AAGAGCTAAGCTGCCAGTTCATTTCTTAGGG
HK_MIA2	ATTTTGCCCCTGGTTCTTCCACCTTGTTGGCCAACAAGCCGATAGAAAAGTTTCAGAGC<br>GAGACGTGCCTGCAGGATACGTCTCAGAAACATGGCACTAACTATAAGAGAAAAGTTTA<br>AGAGCTAAGCTGCCAGTTCATTTCTTAGGG
HK_HMGB3	ATTTTGCCCCTGGTTCTTCCACCTTGTTGGGGCCGCAGCCGGGAACCCAGTTTCAGAG<br>CGAGACGTGCCTGCAGGATACGTCTCAGAAACATGGCGGGAACCCAGGGCCCCCGGT<br>TTAAGAGCTAAGCTGCCAGTTCATTTCTTAGGG
HK_NRP1	ATTTTGCCCCTGGTTCTTCCACCTTGTTGGAGACACCCGGACCTCCCCTGTTTCAGAGC<br>GAGACGTGCCTGCAGGATACGTCTCAGAAACATGGTGGCGCCCAGGGGAGGTCCGTT

	TAAGAGCTAAGCTGCCAGTTCATTTCTTAGGG
HK_CLIC6	ATTTTGCCCCTGGTTCTTCCACCTTGTTGGGCCGCGGAGGTGCAGTGCAGTTTCAGAG CGAGACGTGCCTGCAGGATACGTCTCAGAAACATGGGCGGGCCGGGCACTTCCAAGT TTAAGAGCTAAGCTGCCAGTTCATTTCTTAGGG
HK_ALDH2	ATTTTGCCCCTGGTTCTTCCACCTTGTTGGAGGCGGGGCCCGAAGCGGGGTTTCAGAG CGAGACGTGCCTGCAGGATACGTCTCAGAAACATGGGCAGCGCGCAACATCGCAGGT TTAAGAGCTAAGCTGCCAGTTCATTTCTTAGGG
HK_SCOC	ATTTTGCCCCTGGTTCTTCCACCTTGTTGGTGAGGCGCCGGGAGAAGGAGTTTCAGAG CGAGACGTGCCTGCAGGATACGTCTCAGAAACATGGGTGTCCCGGCCTGAGGTGTGT TTAAGAGCTAAGCTGCCAGTTCATTTCTTAGGG
5_NT	ATTTTGCCCCTGGTTCTTCCACCTTGTTGGGAGTCGGGTAAATAGACAAGTTTCAGAGC GAGACGTGCCTGCAGGATACGTCTCAGAAACATGGGAGTCGGGTAAATAGACAAGTTT AAGAGCTAAGCTGCCAGTTCA
9_NT	ATTTTGCCCCTGGTTCTTCCACCTTGTTGGCCTTGGCTAAACCGCTCCCGTTTCAGAGC GAGACGTGCCTGCAGGATACGTCTCAGAAACATGGCCTTGGCTAAACCGCTCCCGTTT AAGAGCTAAGCTGCCAGTTCA
14_NT	ATTTTGCCCCTGGTTCTTCCACCTTGTTGGGAGGACGATCGTACTCCAGGTTTCAGAGC GAGACGTGCCTGCAGGATACGTCTCAGAAACATGGGAGGACGATCGTACTCCAGGTTT AAGAGCTAAGCTGCCAGTTCA
32_NT	ATTTTGCCCCTGGTTCTTCCACCTTGTTGGGTCAGGTAGAGGGATTGAGGTTTCAGAGC GAGACGTGCCTGCAGGATACGTCTCAGAAACATGGGTCAGGTAGAGGGATTGAGGTTT AAGAGCTAAGCTGCCAGTTCA
59_NT	ATTTTGCCCCTGGTTCTTCCACCTTGTTGGTATGGAGGGCTGGATCTGCGTTTCAGAGC GAGACGTGCCTGCAGGATACGTCTCAGAAACATGGTATGGAGGGCTGGATCTGCGTTT AAGAGCTAAGCTGCCAGTTCA

Table 5: CRISPRi guide RNA oligonucleotides and control sequences.

qPCR Primer	Sequence
TXNIP_pre_qPCR_F	5’ - TTCTATCCTGGGCTGCAAC
TXNIP_pre_qPCR_R	5’ - TCTGGAGAAACAAGACAGCTG
TXNIP_qPCR_F	5’ - GAATACATGTTCCCGAATTGTGG
TXNIP_qPCR_R	5’ - ACTTCTGAGTCAGCACCTTG

Table 6: TXNIP primers for quantitative PCR of expression levels.
